# From Routine Blood Tests to Metabolomics: A Contextual Framework for Interpreting Biomarkers of Training Load, Recovery, and Metabolic Stress in Athletes

**DOI:** 10.3390/metabo16070483

**Published:** 2026-07-09

**Authors:** Mario Muñoz-López, Gonzalo Quesada-Fernández, Edgar Simón Sancho-Haro, Xabier Ramírez de la piscina-Viúdez, Eneko Baz-Valle, José Francisco López-Gil, José Francisco Tornero-Aguilera

**Affiliations:** 1 Department of Sport Sciences, Faculty of Sport and Health Sciences, Fit Generation Research Institute, AD500 Andorra la Vella, Andorra; mario.mlopez@fitgeneration.es (M.M.-L.); xabier.ramirezpiscina@gmail.com (X.R.d.l.p.-V.); enekowushu@gmail.com (E.B.-V.); 2 Department of Nutrition and Dietetics, Faculty of Sport and Health Sciences, Fit Generation Research Institute, AD500 Andorra la Vella, Andorra; gonzalo@fitgeneration.es (G.Q.-F.); edgar.sancho.haro@gmail.com (E.S.S.-H.); 3 School of Medicine, Universidad Espíritu Santo, Samborondón 092301, Ecuador; josefranciscolopezgil@gmail.com; 4 Faculty of Health Sciences, Universidad Autónoma de Chile, Temuco 4780000, Chile

**Keywords:** athletes, biomarkers, exercise metabolism, metabolomics, training load, recovery, metabolic flexibility, muscle damage, inflammation, sports nutrition, iron metabolism, endocrine stress

## Abstract

**Highlights:**

**What are the main findings?**

- Biomarkers in athletes are best interpreted as longitudinal, context-dependent signals rather than standalone diagnostic thresholds.
- Routine blood markers and emerging metabolomics provide complementary information when integrated with training load, nutrition, recovery, symptoms, sport-specific demands, and objective performance.

**What are the implications of the main findings?**

- Athlete biomarker monitoring should prioritize individual baselines, standardized sampling, trend interpretation, and functional triangulation rather than population reference ranges alone.
- Metabolomics may improve biological interpretation of exercise adaptation and maladaptation, but applied decisions should remain cautious until sport-specific validation and incremental value over simpler tools are demonstrated.

**Abstract:**

**Background:** Biomarkers are increasingly used in sport science and sports medicine to monitor training load, recovery, metabolic stress, nutritional status, and potential clinical risk in athletes. However, their interpretation is often limited by overreliance on isolated values, population reference ranges, and simplified thresholds. This narrative review aims to provide a contextual and metabolically informed framework for interpreting routine and emerging biomarkers in athletes. **Methods:** A critical narrative synthesis was conducted across key physiological domains relevant to athlete monitoring, including exercise intensity, metabolic flexibility, muscle damage, protein catabolism, hydration, hematological and iron status, micronutrient and bone–muscle health, inflammation, endocrine stress, sport-specific interpretation, and emerging metabolomics. The review integrated routine laboratory markers with pathway-level metabolomic interpretation and practical decision-making principles. **Results:** Routine markers such as lactate, creatine kinase, urea/blood urea nitrogen (BUN), creatinine, electrolytes, ferritin, C-reactive protein, cortisol, testosterone, and vitamin D are useful only when interpreted in relation to individual baseline, sampling conditions, recent workload, nutrition, hydration, sleep, illness, sex-specific physiology, and performance. Metabolomics expands interpretation by identifying pathway-level signatures involving glycolysis, β-oxidation, amino acid turnover, purine degradation, ketone bodies, acylcarnitines, bile acids, oxylipins, kynurenine metabolites, and exercise-induced signaling molecules such as lactate, β-aminoisobutyric acid (BAIBA), and N-lactoyl-phenylalanine (Lac-Phe). However, omics-derived markers require careful standardization and validation before routine applied use. **Conclusions:** Biomarkers should refine, not replace, clinical reasoning and athlete monitoring. A BASE framework (Baseline, Analytical standardization, Sport-specific context, and Evidence of functional change) may support more precise and proportionate interpretation of both routine blood tests and emerging metabolomic tools in athletes.

## 1. Introduction

A biomarker can be broadly defined as an objectively measured biological characteristic that reflects a normal biological process, a pathological process, or a biological response to an exposure or intervention [1,2].

In biomedical research, biomarkers may serve descriptive, diagnostic, prognostic, predictive, pharmacodynamic, safety, or monitoring purposes; however, their validity depends not only on analytical accuracy, but also on whether the measured characteristic meaningfully represents the biological construct or clinical state of interest [1,2]. This distinction is particularly important in sport and exercise science, where the same biomarker may reflect physiological adaptation, transient perturbation, maladaptation, or disease depending on the timing of sampling, the athlete’s training history, the exercise stimulus, and the broader recovery context [3,4].

In athletes, this conceptual distinction becomes especially important because biomarker interpretation differs from conventional clinical interpretation in at least one fundamental way: exercise itself is both a physiological stimulus and a major preanalytical variable. This is particularly relevant in training-load monitoring because the same external workload may elicit different internal responses according to athlete characteristics, recent training history, and recovery context [5,6]. Recent training can also alter muscle enzymes, inflammatory markers, renal function markers, electrolytes, hormones, metabolites, and plasma volume [7,8]. Consequently, a value outside a general population reference interval may represent an expected sport-related response, whereas a value within the reference interval may still be abnormal for a given athlete if it deviates meaningfully from their usual baseline [7,8].

This problem is evident across multiple biomarker domains. High creatine kinase after eccentric loading, sprinting, collisions, or unaccustomed resistance exercise may reflect expected muscle membrane disruption rather than injury or overtraining [9]. Inflammatory markers may rise transiently after intense or prolonged exercise as part of tissue remodeling, but persistent or marked elevations with systemic symptoms may indicate infection, injury, or insufficient recovery [10]. Similarly, changes in hemoglobin, hematocrit, ferritin, and iron availability may reflect plasma volume expansion, iron deficiency, inflammation, or altered oxygen transport capacity depending on the athlete and context [11].

This interindividual variability is also relevant to injury and illness risk. International consensus statements emphasize the need to manage training and competition load while acknowledging that no single marker captures the full complexity of athlete adaptation or maladaptation [12,13]. Together, these factors limit interpretation based on isolated values or population reference intervals alone. Population ranges remain clinically useful, but they may miss meaningful within-athlete deviations or classify expected exercise-related responses as abnormal. Analytical precision is therefore insufficient when sampling conditions, expected kinetics, and the athlete’s prior trajectory are ignored [4,7,14,15]. The practical challenge is to determine whether a measured change is biologically plausible, methodologically comparable, temporally meaningful, and consistent with the athlete’s broader functional state.

A further controversy concerns the relative value of objective versus subjective monitoring. Athlete-reported measures can be sensitive to perceived load, fatigue, sleep quality, soreness, psychological stress, and general well-being, and in some training-monitoring contexts they may detect changes that are not captured by isolated objective measures [16]. However, this should not be interpreted as a generic superiority of self-report over biomarkers. For clinical, biochemical, or safety-related questions (such as iron restriction, electrolyte disturbance, severe muscle damage, endocrine–metabolic suppression, or Relative Energy Deficiency in Sport (REDs) risk) objective markers may provide information that self-report cannot capture [4,11,15,17]. The most defensible approach is therefore integrative: subjective measures should complement, rather than replace, biomarkers, performance measures, symptoms, recovery, sleep, nutrition, and training load [16].

This limitation of isolated biomarker interpretation provides the rationale for incorporating metabolomics into athlete monitoring. Precisely because the same alteration in a routine biomarker can arise from multiple biological causes, the next interpretive step is not simply to measure more isolated analytes, but to place them within broader pathway-level patterns. Traditional athlete monitoring has relied mainly on selected biochemical, hematological, hormonal, and inflammatory markers [3,4]. These markers remain useful because they are accessible, clinically familiar, and feasible in applied sport settings. However, they offer only a partial view of exercise biology. Exercise simultaneously modifies glycolysis, glycogenolysis, β-oxidation, amino acid turnover, tricarboxylic acid cycle flux, redox balance, purine metabolism, immune signaling, endocrine regulation, vascular function, and interorgan metabolite exchange [18,19]. Metabolomics is therefore not introduced here as a replacement for routine blood testing, but as a complementary strategy to interpret routine markers within dynamic metabolic networks.

In practical terms, metabolomics enables the simultaneous measurement of many low-molecular-weight metabolites that reflect ongoing metabolic activity [20,21]. Acute exercise produces coordinated plasma signatures involving lactate, pyruvate, amino acids, fatty acids, acylcarnitines, ketone bodies, purine metabolites, and tricarboxylic acid (TCA)-cycle intermediates [18]. Multi-omic studies further show that exercise triggers complex, time-dependent responses across metabolic, inflammatory, cardiovascular, and immune pathways [19]. These findings support a shift from interpreting biomarkers as isolated analytes toward understanding them as components of dynamic metabolic networks [20,21].

This shift is especially relevant because the same routine biomarker can arise from multiple biological processes. Elevated urea may reflect high training volume, increased amino acid oxidation, high protein intake, energy deficit, or dehydration; elevated creatinine may reflect renal stress, muscle mass, recent exercise, creatine supplementation, or hemoconcentration; and elevated C-reactive protein may reflect infection, tissue damage, low-grade inflammation, or recent competition [7,8]. Emerging metabolomic approaches may help identify pathway-level patterns consistent with adaptation, substrate stress, inflammation, low energy availability, or impaired recovery, although their applied use still requires stronger validation and careful standardization [20,22].

Lactate illustrates this conceptual transition. Once viewed mainly as a by-product of anaerobic glycolysis, lactate is now recognized as a major energy substrate, gluconeogenic precursor, and signaling molecule involved in intercellular and interorgan metabolic communication [23]. Similarly, exercise metabolomics has identified modality-specific and intensity-dependent responses across endurance and resistance exercise, suggesting that metabolic signatures may eventually help characterize training response, fatigue, recovery, and metabolic flexibility [24]. Nevertheless, metabolomics should complement rather than replace routine biomarkers; otherwise, omics data risk reproducing the same errors of isolated interpretation, insufficient standardization, and premature performance prediction [20,22].

Against this background, the present review addresses a practical and conceptual gap in the field. Previous reviews have often focused on selected laboratory markers, isolated physiological systems, specific clinical problems, or emerging omics technologies. However, fewer reviews have integrated routine blood biomarkers and metabolomics within a single contextual decision framework for sport-specific interpretation. This creates a practical gap: athletes are frequently monitored with accessible markers such as lactate, creatine kinase, urea/blood urea nitrogen (BUN), creatinine, ferritin, C-reactive protein, cortisol, testosterone, and vitamin D, but these values are often interpreted without sufficient integration of baseline variability, sampling conditions, sport demands, nutrition, recovery, symptoms, performance, and pathway-level metabolic biology.

This narrative review aims to address that gap by providing a contextual and metabolically informed framework for interpreting routine and emerging biomarkers of training load, recovery, adaptation, and metabolic stress in athletes. The review focuses on biomarkers related to exercise intensity and metabolic flexibility; muscle damage, mechanical load, and repair; protein catabolism, energy availability, hydration, and renal stress; hematological and iron-related status; micronutrient and bone–muscle health; inflammatory and immunometabolic responses; endocrine stress and recovery; and emerging metabolomic signatures of exercise adaptation. The principal conclusion advanced from the outset is that biomarkers are most useful when they are interpreted as part of a biological and functional decision framework, not as standalone values. Such an approach may improve the practical usefulness of both routine blood tests and metabolomics by linking laboratory data to athlete-specific baselines, sport demands, symptoms, recovery, nutrition, and objective performance.

## 2. Narrative Review Approach

### 2.1. Review Design

This article was designed as a narrative review with a critical and conceptually structured approach. The aim was not to estimate pooled effects, rank interventions, or establish universal diagnostic thresholds, but to synthesize and interpret the literature on athlete biomarker monitoring from a physiological, metabolic, and applied decision-making perspective. Accordingly, the methods aimed to make the narrative selection process transparent without implying the comprehensiveness or reproducibility of a systematic review.

The review was organized around the predefined physiological domains described above rather than around individual biomarkers alone. This structure was selected because the same biomarker may have different meanings depending on timing, sport context, training status, nutritional state, and individual baseline.

### 2.2. Literature Search and Source Selection

A structured literature search was conducted to identify relevant studies, reviews, consensus statements, and methodological papers related to biomarkers, exercise metabolism, metabolomics, training load, recovery, and athlete health. The search was conducted between November 2025 and March 2026, with targeted updates performed before manuscript submission. Because the purpose of this article was conceptual integration rather than estimation of pooled effects, the review was not designed or reported as a systematic review, and no PRISMA flow diagram, formal risk-of-bias assessment, or meta-analysis was performed.

Searches were performed in PubMed/MEDLINE, Scopus, Web of Science, Embase, and Google Scholar. Search terms were combined iteratively using concepts related to the population, context, biomarkers, and physiological domains of interest. Core terms included: “athlete”, “sport”, “exercise”, “training load”, “recovery”, “performance monitoring”, “biomarker”, “blood biomarker”, “metabolomics”, “exercise metabolomics”, “lactate”, “creatine kinase”, “myoglobin”, “lactate dehydrogenase”, “urea”, “blood urea nitrogen”, “creatinine”, “electrolytes”, “sodium”, “potassium”, “hemoglobin”, “hematocrit”, “ferritin”, “transferrin saturation”, “soluble transferrin receptor”, “hepcidin”, “vitamin D”, “C-reactive protein”, “inflammation”, “cytokines”, “cortisol”, “testosterone”, “testosterone-to-cortisol ratio”, “overreaching”, “overtraining”, “relative energy deficiency in sport”, “low energy availability”, “sports nutrition”, “acylcarnitines”, “purine metabolism”, “kynurenine”, “bile acids”, “short-chain fatty acids”, “oxylipins”, “BAIBA”, and “Lac-Phe”.

Source selection was purposive and interpretive. Priority was given to consensus statements, systematic reviews, methodologically informative narrative reviews, mechanistic studies, longitudinal athlete-monitoring studies, and primary studies with direct relevance to exercise metabolism or applied sport settings. Foundational articles were retained when they provided important definitions, methodological principles, or mechanistic frameworks. Studies in non-athletic populations were considered when they directly informed exercise-related mechanisms or interpretive problems relevant to athletes. Sources were retained according to relevance to the review aims, physiological plausibility, methodological informativeness, and contribution to the proposed contextual framework.

### 2.3. Conceptual Synthesis

The synthesis followed a domain-based logic. For each biomarker or biomarker group, the review considered five questions: what the biomarker reflects biologically; why it changes in response to exercise, training, nutrition, recovery, or illness; how it is commonly misinterpreted in athletes; which contextual factors influence its interpretation; and how it relates to broader metabolic pathways or emerging metabolomic signatures.

This approach was chosen because athlete biomarker interpretation is rarely reducible to a single concentration, cut-off, or reference interval. For example, elevated creatine kinase may reflect expected mechanical stress, excessive muscle damage, delayed recovery, or clinical risk depending on symptoms, performance loss, timing, and magnitude of change. Similarly, elevated urea may reflect training volume, high protein intake, dehydration, low energy availability, or increased amino acid oxidation. Therefore, the synthesis emphasized physiological context rather than isolated classification as “normal” or “abnormal”.

### 2.4. Scope of the Review

This review focuses on adult and adolescent athletes engaged in structured training or competitive sport. The term “athlete” is used broadly, including endurance athletes, strength and power athletes, team-sport athletes, combat and contact-sport athletes, aesthetic or weight-sensitive sport athletes, and highly active individuals exposed to systematic training loads. Where relevant, sex-specific considerations, sport-specific demands, and nutritional factors are discussed.

The review does not aim to provide clinical diagnostic criteria, universal biomarker cut-offs, return-to-play rules, or laboratory reference intervals for all athletic populations. Such thresholds are difficult to generalize because biomarker responses vary according to training status, sport modality, sex, age, body composition, nutritional status, environmental conditions, analytical method, and sampling timing. Instead, the review proposes a contextual interpretation framework that prioritizes individual baselines, standardized sampling, longitudinal trends, and triangulation with performance, symptoms, recovery, and training load.

The review also does not treat metabolomics as a replacement for routine blood testing. Rather, metabolomics is considered a complementary layer that may improve biological interpretation by situating accessible but often nonspecific laboratory signals within broader pathway-level patterns. Accordingly, the breadth of biomarker domains covered in this review is deliberate but not intended to be exhaustive: hematological, renal, inflammatory, endocrine, nutritional, and muscle-damage markers are included primarily where they illustrate how routine measurements may acquire different meanings according to athlete-specific context and where pathway-level metabolic or metabolomic evidence may add interpretive context. Detailed diagnostic or disease-specific management falls outside the scope of the review. 

### 2.5. Structure of the Review

The review follows a progressive architecture from conceptual principles to applied interpretation. Section 3 establishes the core principles required for contextual biomarker interpretation. Section 4, Section 5, Section 6, Section 7, Section 8, Section 9 and Section 10 then examine major physiological domains using routine biomarkers as accessible entry points while incorporating pathway-level metabolic and metabolomic evidence where relevant. Section 11 marks the transition to systems-level interpretation by focusing on emerging metabolomics, interorgan signaling, multimodal integration, and methodological constraints. Section 12 applies this integrative logic to sport-specific demands, whereas Section 13 translates the preceding concepts into a practical framework for proportional decision-making.

This progression is intended to move from domain-level biological signals toward increasingly integrative interpretation: first establishing what a biomarker may reflect, then situating signals within broader metabolic systems, next considering the demands and exposure history of the sport, and finally translating the total evidence pattern into action. The intended outcome is therefore not an exhaustive catalogue of biomarkers, but a framework linking biological measurement to context, function, and decision-making.

## 3. Principles for Contextual Biomarker Interpretation in Athletes

Biomarker interpretation in athletes requires a different logic from conventional single-test clinical interpretation. In many clinical contexts, a laboratory result is interpreted primarily by comparison with a population-derived reference interval. In sport, however, the same value may reflect normal adaptation, acute training perturbation, incomplete recovery, nutritional strain, illness, injury, or analytical noise depending on the athlete’s baseline, sampling conditions, recent workload, recovery status, and functional state [4,25]. Therefore, the practical question is often not whether a value falls inside or outside a laboratory range, but whether it is meaningfully different for that athlete, under comparable conditions, and whether it converges with other signs of altered function [14,26]. The same logic applies to routine and metabolomic data: greater analytical dimensionality does not compensate for poor standardization or weak functional anchoring [20,22].

**Figure 1:**
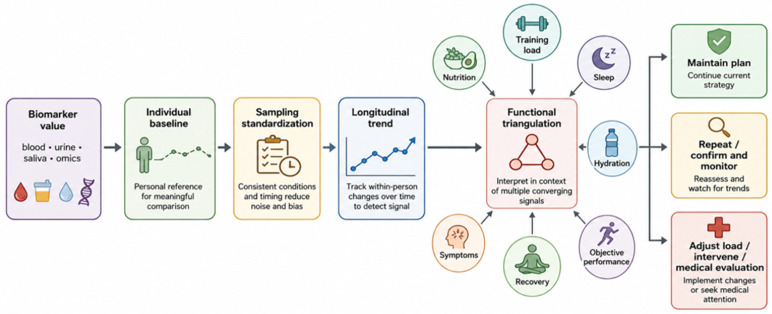
Contextual framework for athlete biomarker interpretation. Biomarker interpretation in athletes should proceed from the measured value toward individualized and contextualized decision-making. The framework emphasizes four sequential filters: individual baseline, sampling standardization, longitudinal trend, and functional triangulation. The final interpretation should integrate training load, nutrition, sleep, hydration, symptoms, recovery, and objective performance before informing whether to maintain the current plan, repeat or confirm the finding, or adjust training and seek further evaluation.

This section proposes four core principles for athlete biomarker interpretation (Figure 1): individual baseline, sampling standardization, longitudinal trend, and functional triangulation. Although presented sequentially, these principles are interdependent rather than isolated. First, the athlete’s usual biological range should be characterized; second, repeated measurements should be collected under sufficiently comparable conditions; third, the magnitude and direction of change should be evaluated over time and against the athlete’s training and competition history; and fourth, interpretation should depend on triangulation with symptoms, recovery, internal load, and objective performance [4,25]. In this structure, longitudinality is not confined to serial laboratory values, and triangulation is not a final binary check: both connect the biomarker trajectory to the athlete’s evolving sport context. This conceptual architecture is later operationalized in the BASE model.

### 3.1. Individual Baseline Versus Population Reference Ranges

Athlete-specific baselines are often more informative than population reference intervals. Population intervals are useful for identifying values that are unusual in general clinical populations, but they are poorly suited to detect meaningful within-athlete change when biological variation is high or training alters resting physiology [14,25]. A biomarker may remain inside the laboratory reference range while still representing a relevant deviation from the athlete’s usual state. Conversely, a value outside the population range may be expected after specific training stimuli, at a certain phase of the season, or in a particular sport discipline [4,26].

This distinction is central to blood profiling in high-performance sport. Pedlar and colleagues emphasized that serial biomarker monitoring can support individualized interpretation for nutrition and physiology, but only when practitioners understand the limitations of one-off testing and the importance of within-athlete change [25]. This is consistent with the reference change value concept, which estimates whether the difference between two serial results is likely to exceed expected analytical and biological variation [26]. In athletes, the same principle applies: change over time should be judged against expected variability, not only against a static group-based interval.

Individualized interpretation is especially important for markers with large interindividual variability, such as creatine kinase, urea, inflammatory markers, and endocrine variables [4,14]. Creatine kinase illustrates this problem clearly: some athletes show large post-exercise increases after eccentric or impact loading, whereas others show smaller responses to similar external work. If the same threshold is applied to all athletes, high responders may be repeatedly misclassified as abnormal, while low responders may appear reassuring even when functionally impaired [9,27]. Individualized reference ranges may therefore improve the detection of relevant deviations in muscle recovery compared with group-based approaches [27,28].

A practical baseline should be built from repeated samples collected during relatively stable training periods, under standardized conditions, and interpreted alongside normal performance and recovery status. Even a small number of well-standardized baseline measurements is usually superior to interpreting a single value against a generic population range [25,27]. However, baselines should be dynamic rather than permanent. They may require recalibration after injury, altitude exposure, major training blocks, weight-loss phases, menstrual-cycle changes, aging, or changes in body composition [4,25]. This is particularly relevant for hematological, endocrine, nutritional, and inflammatory markers, whose interpretation may be influenced by season phase, competition density, travel, and energy availability [11,17].

### 3.2. Standardization of Sampling Conditions

Before a biomarker can be interpreted longitudinally, sampling conditions must be sufficiently comparable. Exercise itself is a powerful preanalytical variable capable of altering muscle enzymes, renal markers, inflammatory proteins, leukocyte counts, hormones, metabolites, plasma volume, and electrolytes [7,8]. Without controlling or documenting recent exercise exposure, a normal response to the last session may be misinterpreted as maladaptation, illness, or poor recovery.

Sampling time is particularly important for biomarkers with circadian or diurnal variation. Cortisol and testosterone are obvious examples, but hematological, metabolic, and inflammatory variables may also vary according to time of day, sleep, feeding, hydration, posture, and prior activity [7,14]. Repeated measurements should therefore be collected at the same time of day, under similar nutritional and hydration conditions, after comparable rest from intense exercise, and using the same laboratory method when possible [4,25].

The minimum contextual information accompanying a biomarker sample should include time of collection, fasting or fed state, hydration status or recent body-mass change, sleep duration and quality, recent training load, time since last intense session or competition, medication and supplement use, illness symptoms, menstrual-cycle context when relevant, travel, heat or altitude exposure, and unusual psychological stressors [4,7]. This information determines whether the biomarker can be interpreted at all. A cortisol value collected after poor sleep and travel is not directly comparable with one collected after a rest day at home; similarly, a creatine kinase value collected after downhill running is not equivalent to one collected after a low-load technical session.

Standardization also requires attention to analytical reliability. Measurement error and within-subject biological variability can obscure meaningful changes or generate false alarms [26,29]. A biomarker with poor analytical precision, high day-to-day variability, or unclear exercise-response kinetics may be scientifically interesting but practically weak for monitoring [29,30]. In applied sport, a standardized but less frequent biomarker protocol may be more valuable than frequent unstandardized testing. The goal is not maximal measurement, but interpretable measurement.

### 3.3. Longitudinal Trends Versus Isolated Values

Sustained trends are more informative than isolated values, but longitudinal interpretation requires more than repeated laboratory measurements alone. Biomarker trajectories should be considered in relation to the athlete’s evolving history of training, competition, recovery, and other relevant exposures. Many biomarkers show transient responses to acute stimuli: creatine kinase may peak 24–72 h after muscle-damaging exercise; inflammatory markers may rise after prolonged or intense exercise; cortisol may increase after sleep restriction, travel, energy deficit, or psychological stress; and urea may rise during high-volume endurance blocks, high protein intake, dehydration, or low energy availability [4,8]. A single altered value may therefore be expected and physiologically appropriate depending on when it occurs within that exposure history.

Longitudinal interpretation helps distinguish expected perturbation from persistent or contextually discordant change. A transient creatine kinase increase after an eccentric strength session may indicate mechanical stress and remodeling, whereas repeated high values accompanied by soreness, reduced range of motion, and performance loss may suggest incomplete recovery or excessive muscle damage [9,10]. Similarly, a single morning cortisol elevation is rarely actionable, but a sustained upward trend together with poor sleep, high perceived exertion, mood disturbance, and declining performance may indicate excessive total stress load [15,31]. The same biomarker trajectory may also carry different implications during a heavy training block, taper, congested competition period, altitude exposure, or return from injury, particularly when it differs from the athlete’s previous response to comparable circumstances.

Trend interpretation should consider direction, magnitude, kinetics, and contextual consistency. A small change may be statistically detectable but practically irrelevant, whereas a larger change may remain difficult to interpret if within-subject variability is high, sampling conditions changed, or the exposure context is not comparable [26,29]. Concepts such as critical difference, reference change value, smallest worthwhile change, and typical error help avoid overreacting to normal biological variation or overlooking changes that are meaningful for the individual athlete [26,29].

Individualized longitudinal models may improve athlete biomarker interpretation by combining repeated measurements with athlete-specific patterns of variation and response. Hecksteden and colleagues developed an approach to individualize monitoring of muscle recovery using creatine kinase and urea [27], and Barth and colleagues later applied individualized reference ranges in elite badminton players during preparation for competition [28]. Adaptive reference range methods may further support personalized interpretation when repeated data are available [32]. However, detecting change does not establish that an athlete will become injured, ill, overtrained, or underperform. Biomarker trajectories are best interpreted as risk-management signals whose meaning depends on both temporal pattern and exposure context, rather than as deterministic forecasts [4,15].

### 3.4. Triangulation with Performance, Symptoms, and Recovery

Functional triangulation is the synthesis step in which the biomarker signal is interpreted against the evidence generated by the preceding filters rather than treated as an independent fourth check. A finding becomes more actionable when it represents a meaningful deviation from the athlete-specific baseline, was obtained under sufficiently comparable analytical conditions, follows a trajectory that is not readily explained by the athlete’s sport-specific exposure and history, and converges with changes in symptoms, recovery, internal load, or objective performance [4,17,25]. Conversely, discordance among these sources should reduce confidence in a single interpretation and usually favor repeat measurement, observation, or reassessment of context rather than automatic intervention.

This synthesis is essential because most biomarkers used in sport are nonspecific. Elevated C-reactive protein may reflect infection, muscle damage, tissue injury, or recent competition; elevated urea may reflect training volume, dehydration, protein intake, or low energy availability; and low testosterone may reflect sleep restriction, energy deficit, illness, travel, psychological stress, or normal biological variation [4,17]. Triangulation therefore reduces the risk of both false positives and false negatives. It does not eliminate uncertainty; rather, it makes decisions proportional to the convergence, magnitude, and trajectory of evidence. In the applied framework introduced later (BASE), this logic is captured by **E**vidence of functional change: evidence is strongest when the information considered under **B**aseline, **A**nalytical standardization, and **S**port-specific context converges with longitudinal changes in symptoms, recovery, or performance.

Subjective athlete-reported measures should not be dismissed as inferior because they are not biochemical. A systematic review found that subjective self-reported measures often showed greater sensitivity and consistency than commonly used objective measures for monitoring responses to training [16]. Athlete self-report tools can capture perceived fatigue, sleep quality, soreness, mood, stress, and recovery, domains that may change before laboratory markers or performance tests detect a problem [16,33]. The best approach is therefore not biomarkers versus subjective measures, but biomarkers plus subjective and performance data.

Objective performance measures provide another anchor. Depending on the sport, these may include sprint times, jump height, bar velocity, submaximal heart-rate response, heart-rate recovery, power output, pace at a given lactate concentration, repeated-sprint ability, technical execution, match-running metrics, or sport-specific tests [5,34]. A creatine kinase increase without soreness or performance decrement may require observation rather than intervention; by contrast, a moderate biomarker change accompanied by reduced performance, poor sleep, and high perceived exertion may be more actionable than a more extreme isolated value.

Recovery status should also be treated as multidimensional. Consensus recommendations emphasize that recovery and performance require monitoring of both physiological and psychological domains [31]. In athlete biomarker interpretation, this means integrating blood markers with sleep, nutrition, hydration, muscle soreness, mood, psychological stress, travel, and readiness indicators [31]. Table 1 summarizes the core principles that should guide this integrated interpretation.

### 3.5. Decision Logic: Green, Yellow and Red Signals

Contextual interpretation should lead to proportional action. Conceptually, findings can be treated as green, yellow, or red according to the convergence, severity, and trajectory of evidence: green when the pattern is compatible with baseline and expected exposure; yellow when interpretation remains uncertain and repeat measurement or contextual reassessment is warranted; and red when sustained or marked abnormalities converge with symptoms, functional impairment, or relevant clinical risk. This logic is developed operationally in Section 13.3; its purpose is to ensure that action reflects the total evidence pattern rather than a single laboratory value [4,25].

## 4. Biomarkers of Exercise Intensity and Metabolic Flexibility

Exercise intensity is one of the strongest determinants of the acute metabolic response to exercise. As intensity increases, adenosine triphosphate (ATP) turnover rises, phosphocreatine (PCr) availability decreases, glycolytic flux accelerates, lactate and hydrogen ion (H^+^) handling become increasingly important, carbohydrate dependence increases, and mitochondrial oxidative phosphorylation must integrate changing inputs from pyruvate, fatty acids, amino acids, ketone bodies, and tricarboxylic acid (TCA) cycle intermediates. From a biomarker perspective, this means that exercise intensity should not be reduced to a single marker, such as blood lactate, respiratory exchange ratio (RER), or heart rate (HR). Instead, intensity reflects a coordinated metabolic state involving substrates, intermediates, redox balance, ions, transporters, and interorgan metabolite exchange.

This point is particularly relevant for a metabolomics-oriented interpretation of athlete monitoring. Exercise metabolomics has shown that endurance, resistance, high-intensity, and combined exercise stimuli induce partly overlapping but also modality- and intensity-specific metabolic signatures involving amino acids, lipids, acylcarnitines, purine metabolites, organic acids, bile acids, ketone bodies, and glycolytic intermediates [35]. These metabolites do not merely describe energy use; they may reflect metabolic flexibility, mitochondrial function, glycogen availability, substrate partitioning, fatigue mechanisms, recovery kinetics, and training adaptation. At the skeletal muscle level, exercise metabolism depends on the rapid coordination of ATP resynthesis through PCr breakdown, glycolysis, glycogenolysis, β-oxidation, the TCA cycle, and oxidative phosphorylation, with relative pathway contribution changing according to intensity, duration, fiber recruitment, oxygen delivery, and substrate availability [36]. Table 2 summarizes the main metabolite classes discussed in this section and their interpretive risks.

### 4.1. Lactate Thresholds and Exercise Intensity Domains

Blood lactate concentration remains one of the most widely used biomarkers for exercise intensity prescription and endurance performance assessment. During incremental exercise, lactate typically remains close to baseline at low intensities, rises progressively as glycolytic flux and pyruvate production increase, and accumulates more rapidly when production exceeds local and systemic clearance capacity. Lactate threshold concepts have therefore been used to estimate physiological transition points, prescribe training zones, and evaluate endurance adaptation [37].

However, lactate threshold terminology is heterogeneous. Different approaches identify the first rise above baseline, fixed concentrations such as 2 or 4 mmol·L^−1^, maximal distance method (Dmax)-derived thresholds, individual anaerobic thresholds, or the highest intensity associated with metabolic steady state. Although many threshold concepts correlate with endurance performance, they are not interchangeable and should not be treated as equivalent biological events [37]. Small differences in the selected threshold method can lead to different training-zone prescriptions, particularly in highly trained athletes, where physiological transitions may occur over a narrow range of speeds or power outputs.

The maximal lactate steady state (MLSS) is often considered a more physiologically meaningful construct than fixed lactate concentrations because it represents the highest workload at which blood lactate concentration can remain stable over time [38]. Yet MLSS testing is time-consuming, protocol-sensitive, and influenced by exercise modality, muscle mass recruitment, nutrition, prior training, and environmental conditions. Therefore, incremental lactate tests are often used as practical approximations, but their interpretation requires caution. A lactate value of 4 mmol·L^−1^, for example, may correspond to different relative intensities across athletes, sports, training phases, and testing protocols [37,38].

MLSS and critical power or critical speed are often discussed together because both are used to approximate the transition between the heavy and severe exercise-intensity domains. However, they should not be treated as identical constructs. MLSS is defined operationally by the highest constant workload at which blood lactate remains stable, whereas critical power is derived from the hyperbolic relationship between power output and time to task failure and represents the asymptote separating intensities at which physiological responses can stabilize from those at which oxygen uptake (VO_2_) blood lactate, inorganic phosphate, and fatigue-related processes continue to rise until intolerance [38,39]. Thus, MLSS is lactate-kinetic and protocol-dependent, while critical power is performance-tolerance based and linked to the finite work capacity above critical power, W′ [39,40].

In practice, MLSS and critical power may occur at similar intensities in some athletes and protocols, which explains why they are sometimes used interchangeably for training-zone prescription. Nevertheless, discordance can occur because they are estimated using different tests, mathematical assumptions, exercise modalities, stage durations, and criteria for stability or exhaustion [37,38,39,40]. Therefore, the relevant point for biomarker interpretation is not to assume equivalence, but to specify which boundary has been assessed and how. The same absolute lactate concentration may have different implications depending on whether the athlete is exercising below, near, or above the heavy–severe boundary.

Critical power also has practical implications for intermittent high-intensity exercise, where repeated bouts above critical power deplete W′ and recovery periods allow partial reconstitution [40]. This is relevant for team sports, combat sports, racquet sports, and interval training, where metabolic stress is not determined only by average intensity, but also by the distribution of work above and below critical thresholds. In such contexts, lactate may reflect the accumulated balance between glycolytic flux, clearance, active recovery, buffering, and repeated recruitment of high-threshold motor units rather than a simple “anaerobic” state [40].

**Figure 2:**
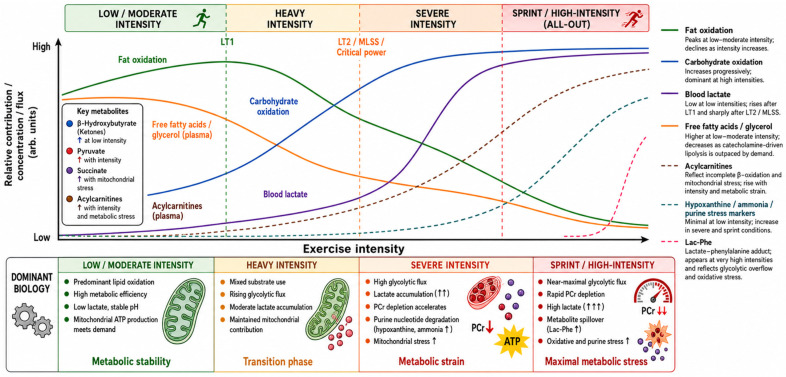
Exercise intensity domains and substrate-dependent metabolic responses. Relative changes in major metabolic pathways and circulating biomarkers across increasing exercise intensity are illustrated schematically, including fat oxidation, carbohydrate oxidation, blood lactate, free fatty acids/glycerol, acylcarnitines, purine stress markers, and Lac-Phe. The figure also summarizes the dominant metabolic biology characterizing the low/moderate, heavy, severe, and sprint/high-intensity domains. Curves are conceptual and intended to represent general physiological patterns; actual responses may vary according to training status, exercise modality, nutritional state, and sampling conditions. Solid and dashed colored lines identify the trajectories of the metabolic pathways and biomarkers according to the color-coded legend: green, fat oxidation; blue, carbohydrate oxidation; purple, blood lactate; orange, free fatty acids/glycerol; brown dashed, acylcarnitines; teal dashed, hypoxanthine/ammonia/purine stress markers; and pink dashed, Lac-Phe. Vertical dashed lines indicate approximate physiological transition points between intensity domains, including LT1, LT2/MLSS/critical power, and the transition toward sprint/all-out exercise. Upward arrows indicate an increase in the corresponding metabolite, pathway activity, or physiological stress, whereas downward arrows indicate depletion or reduction, as exemplified by the progressive decline in phosphocreatine (PCr) availability at severe and sprint/high intensities. Repeated arrows (e.g., ↑↑ or ↑↑↑) denote progressively greater relative increases rather than exact quantitative changes. Green-to-red domain color progression represents the transition from metabolic stability and predominant oxidative metabolism toward increasing glycolytic strain, metabolite accumulation, phosphagen depletion, and maximal metabolic stress.

These exercise-intensity domains and the corresponding shifts in substrate use and metabolite appearance are summarized in Figure 2.

### 4.2. Lactate as Fuel, Shuttle, and Signaling Molecule

The traditional interpretation of lactate as a waste product of anaerobic glycolysis is now obsolete. Lactate is better understood as a central metabolite that links glycolytic and oxidative tissues, contributes to carbohydrate redistribution, supports gluconeogenesis, and participates in intercellular and interorgan communication [23]. During exercise, lactate production reflects high glycolytic flux and rapid nicotinamide adenine dinucleotide, oxidized form (NAD^+^)/nicotinamide adenine dinucleotide, reduced form (NADH) turnover, but its accumulation in blood depends on the balance between production, transport, oxidation, gluconeogenesis, and distribution across active and inactive tissues.

The lactate shuttle concept provides a useful framework for athlete biomarker interpretation. Lactate produced in glycolytic fibers can be transported through monocarboxylate transporters, particularly monocarboxylate transporter 4 (MCT4) in more glycolytic contexts and monocarboxylate transporter 1 (MCT1) in more oxidative contexts, and then oxidized in adjacent muscle fibers, heart, brain, or other tissues [23]. Lactate can also serve as a precursor for hepatic and renal gluconeogenesis through the Cori cycle. Thus, a rise in blood lactate during exercise does not simply indicate “oxygen lack”; it represents the integrated result of glycolytic rate, mitochondrial oxidative capacity, lactate transport, blood flow distribution, and clearance kinetics [23].

Training status strongly modifies lactate interpretation. Endurance-trained athletes may show lower lactate concentrations at the same absolute workload because of improved mitochondrial density, capillarization, oxidative enzyme activity, lactate transport capacity, and substrate utilization. However, at the same relative intensity or near maximal effort, highly trained athletes may still achieve substantial lactate values. Therefore, lactate should be interpreted relative to workload, power output, speed, rating of perceived exertion (RPE), heart rate, substrate oxidation, and prior testing history rather than as an isolated concentration [37].

Lactate is also increasingly relevant as a signaling molecule. Exercise-induced lactate may influence mitochondrial biogenesis, angiogenesis, substrate metabolism, appetite regulation, immune signaling, and redox-sensitive pathways [23]. Although many of these mechanisms remain incompletely translated into applied sport monitoring, they reinforce the idea that lactate is a metabolite with both energetic and signaling relevance, not simply a marker of fatigue.

### 4.3. Glucose, Free Fatty Acids, Ketone Bodies, and Substrate Use

Metabolic flexibility refers to the capacity to adapt fuel selection to changes in energy demand, nutrient availability, and physiological state [41]. In exercise, this involves shifting between lipid oxidation, carbohydrate oxidation, ketone body use, amino acid contribution, and endogenous substrate stores according to intensity, duration, training status, and nutritional context. In athletes, metabolic flexibility is not equivalent to maximizing fat oxidation. Rather, it reflects the capacity to use the appropriate substrate at the appropriate time: preserving carbohydrate at low-to-moderate intensities, rapidly increasing carbohydrate oxidation when high ATP flux is required, oxidizing lactate efficiently, and restoring metabolic homeostasis during recovery [41].

Whole-body substrate oxidation is commonly estimated through indirect calorimetry using oxygen uptake and carbon dioxide production, although these calculations require assumptions and become less valid during non-steady-state exercise, high-intensity exercise, hyperventilation, and substantial lactate buffering [42]. RER, fat oxidation, carbohydrate oxidation, and lactate responses are informative only when the exercise stage is sufficiently stable, the protocol is standardized, and the athlete’s nutritional state is known [42].

Classic isotope-tracer work demonstrated that carbohydrate and fat metabolism are regulated differently across exercise intensity and duration [43]. As intensity increases, carbohydrate availability and oxidation become progressively more important, while plasma free fatty acid contribution may decline despite high lipolytic drive. At lower intensities, free fatty acids and intramuscular triglycerides can contribute substantially to ATP production, whereas at higher intensities, glycogenolysis and carbohydrate oxidation dominate because they support higher ATP resynthesis rates [43]. This shift is central to interpreting blood glucose, lactate, glycerol, non-esterified fatty acids (NEFA), β-hydroxybutyrate, and acylcarnitines during exercise.

The interaction between carbohydrate and fat metabolism is dynamic and influenced by muscle glycogen, plasma glucose, insulin, catecholamines, carnitine availability, acetyl-CoA, malonyl-CoA, mitochondrial transport, and exercise intensity [44]. High carbohydrate availability supports intense endurance performance, but it may suppress fat oxidation during lower-intensity exercise. Conversely, low carbohydrate availability may increase lipid oxidation and selected cellular signaling responses, but can impair high-intensity performance, training quality, immune function, or recovery when poorly periodized [44,45].

Glycogen availability also interacts with training adaptation. Low-glycogen training strategies may increase selected mitochondrial and oxidative signaling responses, but the translation to performance depends on timing, athlete level, total training quality, and adequate recovery [46]. Thus, high fat oxidation or low lactate during submaximal exercise is not always “better”: it may reflect improved oxidative capacity, but also carbohydrate restriction, glycogen depletion, under-fueling, or inability to produce high glycolytic flux when required [45,46].

Fat oxidation during exercise is influenced by intensity, duration, training status, sex, diet, carbohydrate ingestion, and prior exercise [47]. Maximal fat oxidation (MFO) and the intensity eliciting maximal fat oxidation (FATmax) can characterize metabolic phenotype, but their estimation depends on testing conditions and gas-exchange assumptions and is influenced by nutritional state [42,47]. They should therefore be interpreted as context- and protocol-dependent estimates rather than fixed properties of the athlete.

### 4.4. Acylcarnitines and Mitochondrial Fatty Acid Oxidation

Metabolomics has expanded the interpretation of exercise intensity beyond lactate, glucose, and respiratory gases. A systematic review of human exercise metabolomics studies reported that acute exercise commonly changes lactate, pyruvate, fatty acids, acylcarnitines, ketone bodies, nucleotide-related metabolites, and amino acids across blood, urine, and sweat samples [48]. These findings suggest that exercise intensity and recovery can be monitored as pathway-level perturbations rather than as single-analyte responses [48].

Different analytical platforms capture different portions of the exercise metabolome. liquid chromatography–mass spectrometry (LC-MS) may be particularly useful for lipid species, acylcarnitines, bile acids, and broad untargeted profiling; gas chromatography–mass spectrometry (GC-MS) can be valuable for volatile and derivatized metabolites; and nuclear magnetic resonance (NMR) offers high reproducibility but lower sensitivity for many low-abundance compounds [49]. In athlete monitoring, platform selection is therefore not a technical detail but a determinant of which biological questions can be answered [49].

Environmental conditions also modify metabolic signatures. Davison et al. [50] observed that acute hypoxic exercise was associated with changes in lipid, protein, and purine nucleotide metabolism during exercise and recovery, even when the hypoxia-specific effect was subtle compared with the exercise effect itself. This is relevant for athletes training or competing at altitude because lactate, NEFA, acylcarnitines, hypoxanthine, adenosine, amino acids, and oxidative stress-related metabolites may be influenced simultaneously by intensity, oxygen availability, and recovery timing [50].

Acylcarnitines are particularly informative because they reflect the interface between fatty acid transport, β-oxidation, mitochondrial flux, and incomplete substrate oxidation. Long-chain acylcarnitines such as C16 and C18 species may reflect transport of long-chain fatty acids into mitochondria, whereas medium- and short-chain acylcarnitines may indicate downstream β-oxidation flux or incomplete oxidation. Zhang et al. [51] proposed that exercise-associated acylcarnitine patterns may also reflect branched-chain amino acid catabolism, xenometabolism, and potential signaling to muscle afferent neurons.

From an athlete-monitoring perspective, acylcarnitine profiling may help characterize whether a given stimulus is primarily lipid oxidative, glycolytic, mixed, or associated with incomplete mitochondrial substrate flux. However, interpretation remains exploratory because acylcarnitines are influenced by fasting, diet, carnitine availability, glycogen status, exercise duration, sex, training status, metabolic health, and recovery time [49,51]. 

## 5. Biomarkers of Muscle Damage, Mechanical Load, and Repair

Exercise-induced muscle damage (EIMD) is a common consequence of training and competition, especially when exercise includes eccentric loading, repeated stretch-shortening cycles, collisions, downhill running, sprinting, high-volume resistance exercise, or unaccustomed movement patterns [9,52]. In athletes, EIMD should not be interpreted as synonymous with injury. It represents a continuum ranging from expected mechanical and metabolic perturbation that contributes to adaptation, to excessive structural disruption, impaired recovery, or, in rare cases, exertional rhabdomyolysis [9,10].

The biology of muscle damage begins with mechanical strain, particularly during eccentric contractions, when active fibers are lengthened under tension [53]. Proposed mechanisms include non-uniform sarcomere stretching, cytoskeletal and extracellular matrix disruption, excitation–contraction coupling impairment, intracellular Ca^2+^ accumulation, calpain activation, mitochondrial stress, reactive oxygen and nitrogen species generation, and inflammatory cell recruitment [53,54]. These processes can increase sarcolemmal permeability and promote the appearance of intracellular proteins such as creatine kinase (CK), lactate dehydrogenase (LDH), myoglobin, aspartate aminotransferase (AST), and alanine aminotransferase (ALT) in circulation [9,52].

Inflammation and repair are integral to adaptation. Neutrophils, macrophages, cytokines, satellite cells, fibro-adipogenic progenitors, and extracellular matrix remodeling contribute to regeneration [10,55]. Interpretation is further complicated by the repeated bout effect: subsequent exposure to a similar eccentric stimulus generally produces less soreness, strength loss, CK release, and slower recovery than the initial bout [56], likely through combined neural, structural, excitation–contraction, and inflammatory adaptations [54,56]. Accordingly, athlete monitoring should focus on whether the magnitude and duration of the response are appropriate for the stimulus and compatible with functional recovery. Sex-related physiology may modify the magnitude, timing, and interpretation of muscle-damage responses. Compared with men, women may show different metabolic and inflammatory responses to exercise, partly in relation to 17β-estradiol and its potential effects on substrate metabolism, membrane stability, oxidative stress, and repair [57,58,59]. Some eccentric-exercise studies report broadly comparable muscle disruption but attenuated secondary inflammation in women [58]. Menstrual-cycle phase, hormonal contraceptive use, reproductive status, and sampling timing may further modify CK, soreness, and functional recovery [60]. These factors reinforce that circulating biomarkers and functional outcomes reflect overlapping but non-equivalent processes and should, when possible, be interpreted in sex- and hormone-related context. 

**Figure 3:**
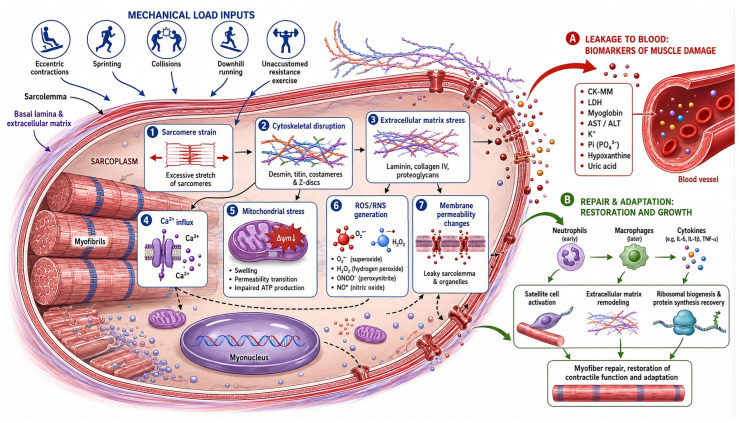
From mechanical load to circulating biomarkers of muscle damage and repair. Mechanical loading stimuli such as eccentric contractions, sprinting, collisions, downhill running, and unaccustomed resistance exercise trigger sarcomere strain, cytoskeletal and extracellular matrix disruption, Ca^2+^ influx, mitochondrial stress, reactive oxygen species (ROS)/reactive nitrogen species (RNS) generation, and membrane permeability changes. These processes promote leakage of muscle-damage biomarkers into the circulation while simultaneously activating inflammatory, reparative, and adaptive pathways that contribute to myofiber restoration and functional recovery. Blue arrows link external mechanical stimuli to the initial cellular impact. Solid black arrows delineate the primary mechanistic sequence of intracellular damage (steps 1–7), while dashed black arrows indicate secondary pathways or internal feedback loops (e.g., calcium-induced mitochondrial stress further compromising membrane integrity). The thick red arrow (**A**) denotes the efflux of intracellular biomarkers through the compromised sarcolemma into the bloodstream, whereas the thick green arrows (**B**) trace the sequential biological responses driving cellular repair and muscular adaptation. Abbreviations: ALT, alanine aminotransferase; AST, aspartate aminotransferase; ATP, adenosine triphosphate; Ca^2^^+^, calcium ion; CK-MM, creatine kinase-MM; IL-1β, interleukin-1 beta; IL-6, interleukin-6; K^+^, potassium ion; LDH, lactate dehydrogenase; Pᵢ (PO_4_^3−^), inorganic phosphate; RNS, reactive nitrogen species; ROS, reactive oxygen species; TNF-α, tumor necrosis factor alpha; ΔΨm, mitochondrial membrane potential.

The sequence linking mechanical load to intracellular stress, biomarker leakage, and subsequent repair and adaptation is summarized in Figure 3.

### 5.1. Creatine Kinase: Utility, Kinetics, and Misinterpretation

CK is the most frequently used blood biomarker of exercise-induced muscle damage. It catalyzes the reversible transfer of a phosphate group between phosphocreatine and adenosine diphosphate (ADP), thereby supporting rapid ATP buffering in tissues with high and fluctuating energy demand [61]. In sport, circulating CK activity is usually interpreted as a marker of skeletal muscle membrane disruption, particularly skeletal muscle creatine kinase isoenzyme (CK-MM), although total CK may also include contributions from cardiac or brain isoforms in specific clinical contexts [61,62].

CK increases after many forms of strenuous exercise, especially eccentric exercise, resistance training, sprinting, downhill running, contact sports, and novel training stimuli [9,61]. Its time course is delayed compared with some other markers: values often rise several hours after exercise, commonly peak between 24 and 72 h, and may remain elevated for several days depending on the magnitude of damage, muscle mass involved, training status, sex, genetics, and recovery conditions [9,57,58,59,60,61]. This delayed kinetic profile makes CK more useful for monitoring recovery after muscle-damaging load than for immediate post-session interpretation.

Sex and hormonal context may contribute to the large interindividual variability observed in CK responses. Differences in muscle mass, substrate metabolism, membrane stability, inflammatory responses, menstrual-cycle phase, and hormonal contraceptive use may modify the magnitude and timing of CK, soreness, and functional loss after eccentric or damaging exercise [57,58,59,60]. Therefore, sex should not be treated simply as a demographic descriptor, but as a relevant biological and interpretive variable when athlete-specific baselines are available.

CK is also frequently misinterpreted. A high value does not automatically indicate injury, overtraining, or a need to stop training [9,61]. Some athletes show chronically higher activity or large post-exercise increases without clinically relevant pathology, whereas others experience soreness or performance loss with only moderate elevations [9,14]. Individual baselines, longitudinal trends, recent exercise, symptoms, and functional status are therefore more informative than generic cut-offs [4,25]. Marked CK elevation accompanied by severe pain, swelling, weakness, dark urine, reduced urine output, electrolyte abnormalities, or rising creatinine should instead prompt concern for a clinically significant process [63,64].

CK is not a direct measure of muscle function. Its association with force loss, delayed-onset muscle soreness (DOMS), range-of-motion restriction, and performance decrement is inconsistent because membrane leakage and clearance kinetics are only partly coupled to excitation–contraction failure, pain, central fatigue, glycogen availability, and neuromuscular performance [9,52,54]. CK should therefore be considered a supportive biomarker rather than a standalone readiness test.

### 5.2. LDH, Myoglobin, and Muscle Membrane Disruption

LDH and myoglobin provide complementary but nonspecific information about muscle membrane disruption and metabolic stress. LDH catalyzes the reversible conversion between pyruvate and lactate, linking glycolysis to redox balance through NADH/NAD^+^ turnover [62]. Because LDH is present in multiple tissues, including skeletal muscle, heart, liver, kidney, and erythrocytes, elevated LDH after exercise may reflect muscle stress but is not tissue-specific [62]. In athletes, LDH is most useful when interpreted together with CK, myoglobin, AST/ALT, symptoms, and recent exercise load.

Myoglobin is an oxygen-binding heme protein located in skeletal and cardiac muscle. It facilitates intracellular oxygen diffusion and may contribute to nitric oxide and reactive oxygen species biology under certain physiological conditions [62]. Following substantial muscle disruption, myoglobin can appear rapidly in blood and urine because of its small molecular size. Compared with CK, myoglobin tends to rise earlier and clear faster, making it potentially useful when the timing of suspected muscle damage is recent [62,63].

The clinical importance of myoglobin lies in its role in exertional rhabdomyolysis and renal stress. Excessive myoglobin release may contribute to renal tubular injury, particularly in the presence of dehydration, heat stress, acidosis, non-steroidal anti-inflammatory drug use, or other nephrotoxic factors [63,64]. In practical terms, dark or cola-colored urine after strenuous exercise should not be dismissed as a normal training response, especially when accompanied by severe pain, weakness, swelling, or systemic symptoms [63,65].

AST and ALT can also rise after strenuous exercise, particularly when muscle damage is substantial [62]. This is important because aminotransferase elevations are often interpreted as hepatic injury in clinical settings. In athletes, isolated AST/ALT elevations after heavy exercise may be muscle-derived, especially when CK and LDH are also elevated and bilirubin, alkaline phosphatase, gamma-glutamyl transferase, and clinical signs do not support liver pathology [62]. Misclassification can lead to unnecessary concern or inappropriate medical conclusions.

Ions and small molecules provide additional mechanistic clues. Muscle membrane disruption and excitation–contraction stress can alter potassium ion (K^+^), sodium ion (Na^+^), calcium ion (Ca^2+^), inorganic phosphate (Pi), hydrogen ion (H^+^) and magnesium ion (Mg^2+^) handling, whereas severe muscle breakdown can contribute to hyperkalemia, hypocalcemia, hyperphosphatemia, hyperuricemia, and renal dysfunction [63,65]. These abnormalities are not typical markers of ordinary EIMD, but they become relevant when the clinical picture suggests rhabdomyolysis or heat-related illness.

### 5.3. DOMS, Performance Loss, and Functional Recovery

Delayed-onset muscle soreness (DOMS) is one of the most recognizable consequences of EIMD, but it is not a biomarker and should not be treated as a direct measure of structural damage [52]. It typically appears several hours after unaccustomed or eccentric exercise, peaks between 24 and 72 h, and resolves over several days depending on the stimulus and athlete adaptation [52,56]. DOMS is influenced by nociceptor sensitization, inflammatory mediators, connective tissue stress, edema, and mechanical disruption, but its magnitude does not necessarily parallel CK, myoglobin, or histological damage [52,55].

Performance loss is often more informative than soreness alone. Eccentric exercise can impair maximal voluntary contraction, rate of force development, jump performance, sprint capacity, range of motion, and sport-specific movement quality [52,54]. These impairments may occur even when soreness is moderate, and they can persist after blood biomarkers begin to normalize [52,54]. Therefore, athlete monitoring should combine biochemical markers with neuromuscular and performance indicators such as countermovement jump, isometric strength, bar velocity, sprint splits, change-of-direction ability, or submaximal power output.

Female athletes may also differ in the relationship between soreness, circulating biomarkers, and functional impairment after muscle-damaging exercise. Sex-related differences in estradiol exposure, inflammatory responses, substrate metabolism, and menstrual-cycle or contraceptive status may influence DOMS, force loss, and recovery kinetics [57,58,59,60]. These factors do not invalidate the general EIMD framework, but they reinforce the need to interpret soreness and performance loss against individualized baselines and, when possible, sex- and hormone-related context.

The repeated bout effect further modifies DOMS and performance responses: after an initial novel eccentric stimulus, subsequent exposure generally produces smaller changes in soreness, CK, and strength loss despite similar external loading [54,56]. This should be considered during preseason, return-to-play, or new eccentric-loading programs. 

Functional recovery should be defined by restoration of relevant task capacity rather than normalization of a blood marker alone [31]. Elevated CK with preserved performance and minimal symptoms may require observation, whereas modest biomarker changes accompanied by strength loss, poor sleep, high RPE, or impaired movement quality may justify recovery support or load adjustment [4,31]. 

### 5.4. Rhabdomyolysis and Clinical Red Flags

Exertional rhabdomyolysis represents the severe end of the muscle-damage continuum and should be distinguished from ordinary EIMD [6,64]. It involves extensive skeletal muscle breakdown with release of intracellular contents, including CK, myoglobin, electrolytes, purines, uric acid, and creatinine, and becomes clinically concerning when accompanied by symptoms, renal stress, electrolyte disturbance, or systemic compromise [63,64,65]. Risk is increased by unaccustomed high-intensity or eccentric exercise, rapid load escalation, heat stress, dehydration, illness, selected drugs or medications, sickle cell trait, and underlying metabolic myopathies [63,64,65].

Mechanistically, failure of muscle energy homeostasis can promote membrane instability, Ca^2+^ overload, mitochondrial dysfunction, protease activation, oxidative stress, and necrosis [65]. ATP depletion impairs ion-pump function, while cytosolic Ca^2+^ accumulation and mitochondrial ROS amplify injury. Thus, rhabdomyolysis is not simply “very high CK” but a systemic risk state involving muscle, kidney, electrolytes, and acid–base homeostasis.

Red flags include severe or worsening muscle pain, marked weakness or swelling, dark urine, reduced urine output, systemic symptoms, hyperkalemia, or rising creatinine [63,64]. Medical evaluation is required when such findings are present. CK thresholds alone are insufficient because clinical severity depends on hydration, heat exposure, renal susceptibility, comorbidities, symptoms, urine findings, and trajectory over time [63,64].

### 5.5. Emerging Metabolomic Signatures of Muscle Damage and Repair

Metabolomics can expand the interpretation of muscle damage beyond enzyme leakage. EIMD is associated not only with CK, LDH, and myoglobin, but also with changes in amino acid metabolism, purine degradation, oxidative stress, lipid remodeling, osmolytes, acylcarnitines, organic acids, and mitochondrial intermediates [35,48]. These pathway-level changes may help distinguish mechanical membrane disruption from metabolic stress, substrate depletion, inflammatory repair, and impaired recovery.

Urinary metabolomics after eccentric exercise has identified changes in metabolites related to energy metabolism, amino acid turnover, organic acids, and purine metabolism, suggesting that EIMD produces systemic metabolic perturbations that extend beyond skeletal muscle enzyme release [66]. Urine may capture downstream products of muscle disruption, nucleotide turnover, oxidative stress, and renal handling, but it is highly sensitive to hydration, diet, timing, renal function, and normalization strategy [48,66].

Purine metabolism is particularly relevant to high-intensity and damaging exercise. Rapid ATP turnover increases ADP and AMP (adenosine monophosphate) accumulation, which can lead to inosine monophosphate formation, adenosine, inosine, hypoxanthine, xanthine, and uric acid production [48,67]. Hypoxanthine and xanthine may reflect adenine nucleotide degradation, whereas uric acid can act as both an antioxidant and a marker of purine catabolism depending on context [67]. In intense or repeated-sprint exercise, elevations in ammonia, hypoxanthine, xanthine, and uric acid may therefore indicate high ATP turnover and metabolic strain rather than structural damage alone [48,67].

Amino acid-related metabolites may also provide insight into muscle remodeling and recovery. Branched-chain amino acids, glutamine, alanine, taurine, histidine-related metabolites, 3-methylhistidine, creatine, creatinine, and urea-cycle intermediates may change after strenuous exercise depending on protein turnover, substrate oxidation, inflammation, and energy availability [35,48]. For example, 3-methylhistidine has been used as an indirect marker of myofibrillar protein breakdown, although interpretation is complicated by diet, meat intake, renal clearance, and whole-body protein turnover. Taurine may reflect osmotic regulation, membrane stabilization, Ca^2+^ handling, antioxidant defense, or muscle stress, but it is not specific enough to diagnose damage.

Lipid-derived metabolites add another layer. Acylcarnitines may increase when β-oxidation flux, incomplete fatty acid oxidation, or mitochondrial substrate overflow rises during and after strenuous exercise [51]. Oxylipins, prostaglandins, leukotrienes, lysophospholipids, and free fatty acids may reflect inflammatory signaling, membrane remodeling, and repair processes [35,68]. In this context, metabolomics may help characterize whether a post-exercise response is dominated by mechanical damage, inflammatory lipid signaling, mitochondrial substrate stress, or combined metabolic load [35,68].

Oxidative stress markers should also be interpreted cautiously. Exercise increases reactive oxygen and nitrogen species, but ROS/RNS are not purely damaging; they participate in redox signaling, mitochondrial biogenesis, antioxidant adaptation, glucose uptake, and muscle remodeling [67]. Excessive oxidative stress may contribute to impaired contractile function and tissue damage, but complete suppression of redox signaling may theoretically interfere with training adaptation. Therefore, oxidative biomarkers are most useful when interpreted as part of a broader pattern including inflammation, muscle function, diet, antioxidant intake, training status, and recovery.

Metabolomics is promising but not yet ready to replace routine markers for muscle damage monitoring. Its greatest value may lie in separating overlapping biological processes: sarcolemmal leakage, energy stress, mitochondrial overload, purine degradation, amino acid turnover, lipid inflammatory signaling, and renal excretion [35,48]. For practical athlete monitoring, the future is unlikely to be a single “muscle damage metabolite”; a more plausible model is a multi-marker pattern combining classical markers such as CK, LDH, myoglobin, creatinine, and electrolytes with selected metabolites such as hypoxanthine, uric acid, ammonia, acylcarnitines, taurine, 3-methylhistidine, oxylipins, and TCA-cycle intermediates [35,48].

## 6. Biomarkers of Protein Catabolism, Energy Availability, and Hydration

Protein catabolism, energy availability, hydration, renal function, and electrolyte balance are tightly interconnected in athletes. High training volume, prolonged exercise, heat exposure, low carbohydrate or energy availability, dehydration, and muscle damage may alter nitrogen handling, amino acid oxidation, creatinine kinetics, renal perfusion, osmolality, and electrolyte concentrations [8,36]. Accordingly, urea/blood urea nitrogen (BUN), creatinine, estimated glomerular filtration rate (eGFR), electrolytes, urine concentration measures, and body-mass change should not be interpreted as isolated markers of “training stress”, “kidney function”, or “hydration status” [4,25]. Urea integrates nitrogen disposal, protein intake, energy availability, and hydration; creatinine reflects muscle turnover, muscle mass, renal filtration, hydration, and recent exercise; and electrolytes reflect fluid balance, renal regulation, sweat losses, and transcellular shifts [69,70,71,72,73]. The central interpretive challenge is therefore to distinguish expected exercise-related responses from under-fueling, dehydration, renal stress, or clinically relevant electrolyte disturbance. 

### 6.1. Urea/BUN: Training Volume, Protein Catabolism, or Dehydration?

Urea is the major end product of amino nitrogen disposal. During amino acid catabolism, ammonia (NH3/NH4^+^) is converted in the liver to urea through a pathway involving ornithine, citrulline, argininosuccinate, arginine, and fumarate, thereby linking nitrogen disposal with mitochondrial and tricarboxylic acid-cycle metabolism [69]. Circulating urea/BUN may rise with increased amino acid oxidation, high protein intake, low energy or carbohydrate availability, dehydration, or altered renal handling [3,8]. Consistent with this complexity, prolonged exercise has been associated with increased serum urea and reduced amino nitrogen [69], but an elevated value should be interpreted as a nitrogen-related signal rather than a specific marker of training load. 

BUN interpretation is especially vulnerable to nutritional confounding. A resistance-trained athlete consuming a high-protein diet may show higher urea production without excessive training stress, whereas an endurance athlete in low energy availability may show elevated urea because amino acids are being oxidized to support energy metabolism [3,74]. In the latter case, urea may be accompanied by weight loss, high perceived exertion, poor recovery, reduced libido or menstrual disturbance, low triiodothyronine, altered resting metabolic rate, or impaired performance, depending on severity and duration [17,75]. Thus, BUN is most meaningful when interpreted alongside energy intake, protein intake, carbohydrate availability, body-mass trend, urine concentration, and training volume.

A further issue is hydration. Hypohydration and hemoconcentration can increase BUN without a proportional increase in protein catabolism [70,71]. Conversely, aggressive fluid intake may dilute BUN and obscure underlying metabolic stress. Therefore, BUN should be interpreted with hydration markers such as plasma osmolality, urine osmolality, urine specific gravity, body-mass change, sweat rate, and sodium losses when available [70,71]. In applied settings, a rising BUN across a training block should prompt questions about fueling and hydration before it is attributed to “overtraining”.

### 6.2. Creatinine: Renal Marker or Muscle-Mass Artifact?

Creatinine is formed from the nonenzymatic conversion of creatine and phosphocreatine, both of which are abundant in skeletal muscle. Because daily creatinine production is influenced by muscle mass, meat intake, creatine supplementation, training status, and recent muscle damage, athletes (especially strength and power athletes) may show higher creatinine values than non-athletic reference populations without true renal disease [7,8]. Creatinine is therefore a useful but imperfect renal marker in sport.

Exercise can acutely increase serum creatinine through reduced renal blood flow, hemoconcentration, dehydration, increased muscle creatine turnover, or substantial muscle breakdown [63,72]. Prolonged exercise and dehydration may also reduce eGFR and increase kidney-stress markers, making post-exercise creatinine difficult to interpret without sampling timing, hydration status, CK, myoglobin, urine findings, and symptoms [72].

Creatinine-based eGFR is particularly problematic in muscular athletes because high endogenous creatinine production or creatine supplementation may lead to underestimation of renal function. Conversely, normal creatinine does not exclude transient tubular stress after prolonged exercise, heat exposure, or hypohydration [72,73]. Urinary kidney injury molecule-1 (KIM-1) and neutrophil gelatinase-associated lipocalin (NGAL) may provide complementary information on tubular injury in research settings [73]. Creatinine should therefore be interpreted as a dual-context marker of renal handling and muscle/exercise exposure, with clinical concern increasing when changes converge with markedly elevated CK, myoglobinuria, reduced urine output, electrolyte abnormalities, or systemic symptoms [63,64].

### 6.3. Sodium, Potassium, and Exercise-Associated Electrolyte Disturbances

Electrolytes are essential for extracellular fluid volume, membrane potential, action potential propagation, excitation–contraction coupling, acid–base regulation, and cardiac rhythm. Sodium (Na^+^) and chloride (Cl^−^) dominate extracellular osmolality, potassium (K^+^) is the major intracellular cation, calcium (Ca^2+^) regulates excitation–contraction coupling and signaling, magnesium (Mg^2+^) participates in ATP-dependent enzymatic reactions, and bicarbonate (HCO_3_^−^) contributes to buffering and acid–base balance [70,71]. During exercise, sweat losses, fluid intake, renal regulation, aldosterone, vasopressin, intracellular–extracellular ion shifts, and acid–base changes all influence circulating electrolytes [70,71].

Exercise-associated hyponatremia is one of the most important electrolyte disorders in endurance sport. Hew-Butler et al. [70] defined exercise-associated hyponatremia as a serum sodium concentration below 135 mmol·L^−1^ during or up to 24 h after physical activity. The main mechanisms include excessive hypotonic fluid intake, impaired free-water excretion, non-osmotic arginine vasopressin secretion, sodium losses, and prolonged exercise duration [70]. Clinically, the key risk is cerebral edema, especially when hyponatremia is symptomatic with headache, confusion, vomiting, altered mental status, seizures, or coma [70].

The interpretation of sodium in athletes must therefore distinguish dehydration from overhydration. A fatigued endurance athlete with dizziness and collapse may be dehydrated, hyponatremic, hyperthermic, hypoglycemic, or experiencing another medical problem. Treating all collapse as dehydration and encouraging excessive fluid intake may worsen hyponatremia [70]. This is a critical example of why biomarkers must be interpreted with context: Na^+^, plasma osmolality, body-mass change, fluid intake history, urine output, symptoms, environmental heat, and event duration all matter.

Potassium also requires context. During high-intensity exercise, K^+^ shifts from contracting muscle into the extracellular space, contributing to membrane depolarization, fatigue, and altered excitability; during recovery, Na^+^/K^+^-ATPase activity helps restore gradients [36]. Mild transient changes are expected during and after intense exercise, but clinically significant hyperkalemia may occur in severe rhabdomyolysis, renal impairment, acidosis, or heat illness [63,65]. Hypokalemia may be associated with gastrointestinal losses, inadequate intake, excessive sweating in some contexts, or inappropriate replacement strategies, and may contribute to weakness, cramps, or arrhythmia risk [70,71].

### 6.4. Low Energy Availability and REDs

Low energy availability (LEA) occurs when dietary energy intake is insufficient to support both exercise energy expenditure and the physiological functions required for health. It is central to Relative Energy Deficiency in Sport (REDs) and may affect endocrine function, bone metabolism, immunity, protein synthesis, hematological status, gastrointestinal function, cardiovascular function, mood, and performance [17,75,76,77]. From a biomarker perspective, LEA is challenging because no single analyte confirms or excludes it. Instead, LEA should be understood as a coordinated state of energy conservation and metabolic reallocation, in which the organism reduces non-essential physiological processes while attempting to preserve short-term energy homeostasis [17,76].

This energy-conservation response has several endocrine–metabolic features relevant to biomarker interpretation. LEA may be associated with reduced resting metabolic rate, lower leptin and insulin signaling, altered thyroid function—particularly reduced triiodothyronine (T3)—suppression of reproductive hormones, changes in cortisol regulation, lower insulin-like growth factor-1 (IGF-1), impaired bone turnover, iron dysregulation, increased injury risk, and altered substrate metabolism [17,75,76]. These changes should not be interpreted as isolated diagnostic markers of LEA, because their magnitude and timing depend on sex, training load, body composition, duration and severity of energy deficit, carbohydrate availability, menstrual or reproductive status, and recent exercise [17,75,76]. Rather, they represent a pattern of adaptive downregulation that becomes clinically relevant when it is sustained, symptomatic, or associated with impaired health or performance.

From a metabolically oriented perspective, LEA is not only a problem of “low calories” but also a shift in substrate availability and pathway prioritization. Low carbohydrate availability and glycogen depletion may increase reliance on lipid oxidation, ketogenesis, gluconeogenesis, and amino acid-derived carbon skeletons. In this context, circulating glucose, β-hydroxybutyrate, free fatty acids, acylcarnitines, amino acids, urea-cycle intermediates, urea/BUN, and cortisol-related signals may reflect the interaction between energy deficit, substrate restriction, training load, and recovery state [45,74,78,79]. Importantly, these metabolites do not diagnose LEA on their own; they provide pathway-level clues that require integration with dietary assessment, body-mass trend, menstrual or reproductive function, symptoms, endocrine markers, bone health, and performance.

LEA may arise intentionally or unintentionally through insufficient fueling, within-day energy deficits, sport-specific physique pressures, disordered eating, or broader environmental influences [75,76,77]. These contexts matter because similar biomarker patterns may have different implications depending on whether the deficit is acute or chronic, intentional or unintended, and accompanied by clinical or performance impairment. Protein metabolism is particularly relevant to LEA. Areta et al. [74] observed that short-term energy deficit reduced resting skeletal muscle protein synthesis, but resistance exercise combined with protein ingestion could rescue aspects of the response. This finding is important for athlete monitoring because low energy availability may alter the interpretation of urea, amino acids, creatinine, 3-methylhistidine, and recovery markers. Elevated urea during heavy training may indicate increased amino acid oxidation, but in the presence of weight loss, poor recovery, reduced performance, menstrual disturbance, low libido, low T3, low leptin, low IGF-1, or recurrent illness or injury, it may point toward inadequate energy availability rather than simply high training load [17,74,75,76].

LEA also interacts with carbohydrate availability. When glycogen and carbohydrate intake are low, amino acids may contribute more to gluconeogenesis and energy production, potentially increasing nitrogen disposal through urea [45,74]. Ketone bodies such as β-hydroxybutyrate may rise during fasting, carbohydrate restriction, rapid weight loss, or prolonged exercise, but this should not be automatically interpreted as superior metabolic flexibility. In an athlete with concurrent fatigue, weight loss, menstrual disturbance, endocrine suppression, poor recovery, or declining performance, elevated β-hydroxybutyrate may instead indicate insufficient energy or carbohydrate availability [17,78,79]. Biomarkers should therefore be used to support a broader assessment of energy availability rather than to replace it.

Emerging metabolomic evidence may help identify early pathway-level signatures of short-term LEA, but this field remains exploratory. Large-scale metabolomics can capture coordinated changes in lipid species, fatty acids, ketone-related metabolites, amino acid metabolism, and other energy-related pathways during controlled LEA exposure [79]. However, these signals are not yet validated as clinical or sport-specific diagnostic thresholds. Their current value is conceptual and mechanistic: they reinforce that LEA should be interpreted as a systemic metabolic state affecting endocrine regulation, substrate selection, protein turnover, bone metabolism, and recovery capacity, rather than as a single abnormal blood value.

### 6.5. Nutritional Confounding in Biomarker Interpretation

Nutritional intake can substantially modify biomarkers used to assess training stress and recovery. Protein and meat intake may alter urea, creatinine, and 3-methylhistidine; creatine supplementation may increase creatinine without kidney injury; carbohydrate restriction may modify glucose, lactate, ketones, and substrate use; and fluid intake may concentrate or dilute plasma and urine markers [7,45]. Biomarker panels should therefore be interpreted with basic information on recent energy and carbohydrate availability, protein intake, supplement use, alcohol intake, and fluid strategy [25,75]. Nutritional confounding is not merely a nuisance variable: it may change the biological meaning of the measured signal. Hydration adds another layer. Body-mass losses, plasma volume shifts, urine concentration, sweat sodium concentration, and fluid intake can all affect interpretation of urea, creatinine, sodium, potassium, chloride, osmolality, and renal biomarkers [70,71,72,73]. Sawka et al. [71] emphasized that exercise fluid replacement should consider sweat rate, electrolyte losses, exercise duration, environmental conditions, and individual variability. In applied settings, the most informative hydration interpretation often comes from combining body-mass change, urine measures, thirst, sweat rate, environmental heat, and performance rather than relying on a single marker [70,71].

For metabolomics, nutritional confounding is even greater. Amino acids, acylcarnitines, ketone bodies, bile acids, organic acids, glucose, lactate, urea-cycle intermediates, and lipid species may all change according to diet composition, timing of feeding, fasting duration, supplement use, and carbohydrate availability [35,48]. Metabolomic samples collected after different pre-test meals, training loads, or hydration states may therefore reflect protocol variation rather than athlete state. Standardization or careful recording of diet and fluid intake is essential for any metabolomics-informed monitoring model [20,21].

## 7. Hematological and Iron-Related Biomarkers

Hematological and iron-related biomarkers are central to athlete monitoring because they connect oxygen transport, erythropoiesis, mitochondrial respiration, immune function, energy metabolism, fatigue, and endurance performance. Hemoglobin (Hb), hematocrit (Hct), red blood cell count, mean corpuscular volume (MCV), mean corpuscular hemoglobin (MCH), ferritin, serum iron, transferrin, transferrin saturation (TSAT), soluble transferrin receptor (sTfR), reticulocyte hemoglobin content, erythropoietin (EPO), and hepcidin-25 reflect different components of oxygen delivery and iron homeostasis [11,80]. These markers are metabolically relevant because iron is required for hemoglobin, myoglobin, cytochromes, iron–sulfur proteins, mitochondrial electron transport, oxidative phosphorylation, and multiple enzymes involved in energy production [81].

Athletes present a particular interpretive challenge because endurance training, altitude exposure, plasma volume expansion, hemolysis, sweating, gastrointestinal blood loss, inflammation, hepcidin responses, menstrual blood loss, dietary restriction, and low energy availability can all modify hematological and iron markers [11,81]. Consequently, low Hb or Hct may reflect true anemia, dilutional pseudoanemia, iron deficiency, inflammation, or recent plasma volume expansion, whereas low ferritin may indicate depleted iron stores even before Hb falls [80,82,83,84]. The central question is not simply whether an athlete is anemic, but whether oxygen transport, iron availability, erythropoiesis, or mitochondrial iron-dependent metabolism are compromised.

From a metabolomics-informed perspective, routine iron markers may also be situated within broader patterns of energy metabolism, inflammatory signaling, oxidative stress, and erythropoietic demand. Such profiles may eventually help distinguish isolated iron depletion from broader states involving low energy availability or inflammatory restriction. However, metabolomics does not currently replace ferritin, TSAT, sTfR, hepcidin, or established hematological assessment, and its incremental value for athlete monitoring remains to be demonstrated [19,35].

### 7.1. Hemoglobin, Hematocrit, and Oxygen Transport

Hemoglobin is the principal oxygen-carrying protein in blood, whereas hematocrit reflects the proportion of blood volume occupied by erythrocytes. In athletes, both are concentration-based measures and therefore depend on red-cell mass and plasma volume [82,83]. This distinction is critical because endurance training may lower Hb concentration and Hct through plasma volume expansion without reducing total hemoglobin mass or oxygen-carrying capacity [82,83]. This phenomenon is central to the concept of sports pseudoanemia.

Altitude further complicates interpretation by stimulating EPO and erythropoiesis while simultaneously altering plasma volume. Hb and Hct are therefore useful but incomplete markers of oxygen transport; when anemia, impaired performance, or altitude adaptation is a concern, interpretation may require ferritin, TSAT, sTfR, reticulocyte indices, inflammatory markers, and, in specialized settings, total hemoglobin mass assessment [11,80]. Reduced oxygen delivery can alter substrate use and endurance capacity, but Hb concentration alone should not be used to infer metabolic function without considering plasma volume, iron status, sex, altitude, hydration, symptoms, and training phase [45,81,82,83].

### 7.2. Plasma Volume Expansion and Sports Pseudoanemia

Plasma volume expansion is a common endurance-training adaptation that supports venous return, stroke volume, thermoregulation, and cardiovascular stability while lowering concentration-based hematological markers through dilution [82]. Consequently, mildly reduced Hb or Hct may represent sports pseudoanemia rather than impaired erythropoiesis [80,83].

Pseudoanemia becomes more plausible when ferritin, TSAT, sTfR, red-cell indices, symptoms, and performance do not indicate iron restriction. Conversely, low Hb or Hct accompanied by low ferritin or TSAT, elevated sTfR, microcytosis, reduced reticulocyte hemoglobin, fatigue, or declining endurance performance increases concern for true iron-restricted erythropoiesis [11,85]. Because plasma volume also varies with hydration, heat acclimation, recent exercise, posture, and sodium intake, serial measurements should be obtained under comparable conditions whenever possible [7,25,82].

### 7.3. Ferritin, Transferrin Saturation, and Soluble Transferrin Receptor

Ferritin is widely used to estimate iron stores in athletes but also acts as an acute-phase reactant and may rise with inflammation, infection, liver stress, or tissue damage [11,84]. Low ferritin strongly suggests depleted stores, whereas normal or elevated values do not exclude functional iron restriction when inflammation is present. This is particularly relevant after heavy training or competition, when CRP and other inflammatory signals may complicate interpretation [11,84]. Athlete-specific ferritin interpretation remains debated, and universal cut-offs should be avoided across sex, sport, altitude exposure, symptoms, and performance context [11,85].

TSAT provides information on circulating iron availability and may be particularly informative when ferritin is ambiguous; serum iron alone is less reliable because of diurnal, dietary, inflammatory, and exercise-related variation [80,84]. sTfR reflects cellular iron demand and iron-restricted erythropoiesis and is generally less affected by inflammation than ferritin, although erythropoietic activity and assay variability remain relevant [86,87]. Combining ferritin with TSAT, inflammatory context, red-cell indices, and, when available, sTfR or reticulocyte hemoglobin provides a more coherent assessment than ferritin alone [11,80,84,85,86,87]. Table 3 summarizes how these markers can be interpreted in athlete monitoring.

### 7.4. Hepcidin, Inflammation, and Exercise-Induced Iron Perturbations

Hepcidin-25 is the master regulatory hormone of systemic iron metabolism. It is produced mainly by the liver and binds to ferroportin, the principal cellular iron exporter on enterocytes, macrophages, and hepatocytes. This interaction promotes ferroportin internalization and degradation, reducing dietary iron absorption and iron release from stores [88]. In athletes, hepcidin is important because exercise-induced inflammation, particularly interleukin-6 (IL-6) signaling, can transiently increase hepcidin and reduce iron availability during recovery [88,89].

Peeling et al. [88] described athletic-induced iron deficiency as a process involving inflammation, cytokines, and hormones, with hepcidin playing a central role. Later, Peeling et al. [89] observed that athletes with different baseline ferritin concentrations showed different post-exercise hepcidin responses, suggesting that iron status modifies the hepcidin response to training. This is highly relevant for athletes with borderline iron stores, who may be repeatedly exposed to post-exercise windows of reduced iron absorption during high-volume training [89].

Domínguez et al. [90] reviewed acute exercise effects on hepcidin and emphasized that exercise can increase hepcidin several hours after a session, often following IL-6 elevation. Larsuphrom et al. [91] systematically reviewed hepcidin changes during aerobic and resistance exercise programs, reinforcing that exercise modality, duration, intensity, and training status may influence responses. Ishibashi et al. [92] observed that twice-a-day endurance exercise elevated serum hepcidin 24 h after exercise in female long-distance runners, suggesting that training frequency and recovery timing may be relevant for athletes at risk of iron deficiency. Nutritional context may also modify the iron–inflammation response to exercise; for example, short-term low carbohydrate availability has been shown to alter iron and immune responses in elite athletes, highlighting that hepcidin-related interpretation should consider both exercise load and fueling strategy [93].

This hepcidin physiology has potential implications for iron intake timing, but practical recommendations should remain provisional. If hepcidin rises in the hours after exercise, iron absorption from meals or supplements may be reduced during that window [88,90]. Although biologically plausible, evidence that such timing strategies meaningfully improve long-term iron status in athletes remains limited [90,93]. Iron supplementation and its timing should therefore be individualized according to documented iron status, dietary intake, gastrointestinal tolerance, menstrual context, inflammation, and medical oversight rather than inferred from exercise timing alone [11,88,89,90,91,92,93].

However, hepcidin measurement is not yet a routine monitoring tool in most sport settings because of assay availability, biological variability, cost, limited applied thresholds, and uncertainty about how best to translate single hepcidin values into training or supplementation decisions [89,90,91]. In applied practice, hepcidin is currently more useful as a mechanistic explanation for exercise-related iron perturbations than as a standalone decision marker.

**Figure 4:**
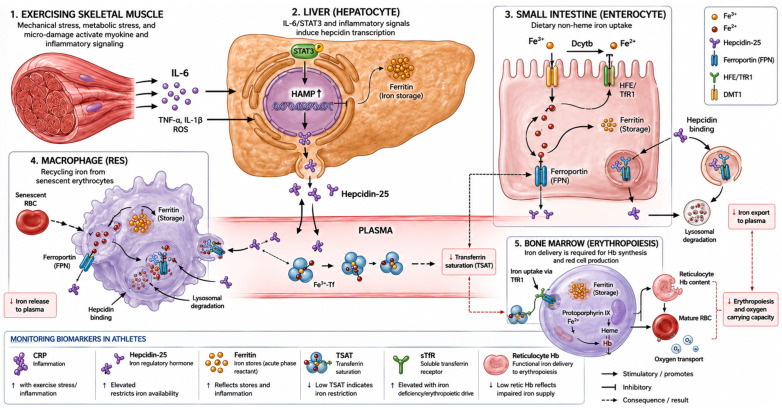
Exercise, inflammation, hepcidin, and iron availability in athletes. Exercise-induced inflammatory and metabolic stress, particularly through IL-6/STAT3 (signal transducer and activator of transcription 3) signaling, stimulates hepatic hepcidin production, which restricts iron export from enterocytes and macrophages via ferroportin internalization and degradation. This reduces transferrin saturation and iron availability for erythropoiesis, thereby affecting oxygen-carrying capacity and adaptation. The figure also summarizes the principal biomarkers used to monitor iron metabolism in athletes, including CRP, hepcidin-25, ferritin, transferrin saturation, soluble transferrin receptor, and reticulocyte hemoglobin. Abbreviations: CRP, C-reactive protein; Dcytb, duodenal cytochrome b; DMT1, divalent metal transporter 1; Fe^2^^+^, ferrous iron; Fe^3^^+^, ferric iron; FPN, ferroportin; HAMP, hepcidin antimicrobial peptide; Hb, hemoglobin; HFE, homeostatic iron regulator protein; IL-1β, interleukin-1 beta; IL-6, interleukin-6; O_2_, molecular oxygen; RBC, red blood cell; RES, reticuloendothelial system; ROS, reactive oxygen species; sTfR, soluble transferrin receptor; STAT3, signal transducer and activator of transcription 3; Tf, transferrin; TfR1, transferrin receptor 1; TNF-α, tumor necrosis factor alpha; TSAT, transferrin saturation.

Inflammation complicates iron interpretation through multiple pathways. IL-6 can increase hepcidin, ferritin may rise as an acute-phase reactant, macrophage iron sequestration may increase, serum iron may fall, and TSAT may decrease despite adequate or elevated ferritin [84,88]. This creates the pattern of functional iron restriction: iron stores may appear present, but iron is not adequately available for erythropoiesis or tissue metabolism [84,86,87]. Figure 4 summarizes this exercise–inflammation–hepcidin pathway and its consequences for iron availability. 

### 7.5. Sex-Specific Considerations and Endurance Athletes

Iron-deficiency risk is heterogeneous across athletes and may be higher in female and adolescent athletes, endurance athletes, athletes in weight-sensitive sports, vegetarians or vegans, those with low energy availability, and athletes exposed to altitude [11,75]. Contributing mechanisms include menstrual blood loss, restricted intake, gastrointestinal or sweat losses, mechanical hemolysis, increased erythropoietic demand, and hepcidin-mediated restriction of iron availability [11,89]. Sport type and sex also influence hematological profiles, supporting context-specific rather than universal athlete thresholds [85,94].

Endurance and altitude settings warrant particular attention because high training volumes, plasma volume expansion, repeated inflammatory exposure, and increased erythropoietic demand may coexist. Athletes entering altitude training with low or borderline iron stores may have insufficient availability to support the expected erythropoietic response [11,88]. Ferritin should therefore be interpreted alongside TSAT, Hb, reticulocyte indices, inflammatory context, dietary intake, symptoms, menstrual status, and performance where relevant [11,85,86,87,88].

Fatigue alone should not trigger iron supplementation because it is nonspecific and may reflect low energy availability, illness, sleep disturbance, overreaching, psychological stress, thyroid dysfunction, or inadequate carbohydrate availability [15,17]. Treatment decisions should be based on biochemical evidence, symptoms, dietary assessment, risk profile, and appropriate clinical oversight; the aim is correction of deficiency or insufficient availability, not indiscriminate supplementation [11,81].

## 8. Micronutrient and Bone–Muscle Health Biomarkers

Micronutrient and bone–muscle health biomarkers are relevant to athlete monitoring because they connect skeletal integrity, muscle function, calcium-phosphate homeostasis, endocrine regulation, energy availability, and injury risk. Among these, 25-hydroxyvitamin D [25(OH)D], calcium, phosphate, parathyroid hormone (PTH), bone-specific alkaline phosphatase (BALP), osteocalcin (OC), procollagen type I N-terminal propeptide (P1NP), C-terminal telopeptide of type I collagen (CTX-I), and sclerostin may help contextualize bone–muscle adaptation and bone stress injury risk [95,96]. However, these markers are not simple performance biomarkers; they are pathway-dependent signals influenced by sun exposure, diet, supplementation, energy availability, mechanical loading, sex hormones, age, season, assay type, and recent exercise [95,96,97].

In a metabolically oriented review, vitamin D and bone biomarkers should not be reduced to a binary deficiency/non-deficiency framework. Vitamin D metabolism involves cutaneous synthesis of cholecalciferol, hepatic 25-hydroxylation to 25(OH)D, renal and extra-renal 1α-hydroxylation to 1,25-dihydroxyvitamin D, and downstream regulation of intestinal calcium and phosphate absorption, PTH secretion, bone mineralization, and muscle function [96]. Bone turnover markers reflect osteoblast and osteoclast activity, collagen formation and degradation, and osteocyte-mediated mechanotransduction, but their interpretation in athletes requires attention to timing, plasma volume shifts, circadian variation, feeding, menstrual status, endocrine context, and recent loading [98].

### 8.1. Vitamin D in Athletes: Muscle, Immunity, and Bone

25(OH)D is the standard circulating biomarker used to assess vitamin D status because it reflects vitamin D input from sunlight exposure, diet, and supplementation more reliably than 1,25-dihydroxyvitamin D. Farrokhyar et al. [95] reported that vitamin D inadequacy is common in athletes, with higher risk in winter, early spring, higher latitudes, and indoor sports. For athlete monitoring, vitamin D status should therefore be viewed as a seasonal, geographic, behavioral, and sport-specific variable, not only as a nutritional marker [95].

Vitamin D has biological relevance for bone and muscle because the vitamin D endocrine system regulates calcium and phosphate homeostasis, skeletal mineralization, and muscle cell function [96]. In muscle, vitamin D receptor-related pathways have been linked to calcium handling, protein synthesis, mitochondrial function, and neuromuscular performance, although performance responses to supplementation remain inconsistent and appear strongest when deficiency is present [95,96]. Thus, 25(OH)D is best interpreted as a health and adaptation-support marker rather than as a direct performance predictor.

Vitamin D status is also relevant to bone stress injury. Knechtle et al. [97] reviewed the relationship between vitamin D and stress fractures in sport and emphasized that low 25(OH)D may contribute to impaired calcium homeostasis and bone vulnerability, although stress fractures are multifactorial. In athletes, low 25(OH)D should be interpreted together with calcium intake, PTH, phosphate, menstrual or endocrine status, energy availability, training load, previous bone stress injury, and sport-specific loading pattern [97].

A key challenge is that general clinical thresholds may not fully capture sport-specific concerns. Concentrations below 50 nmol·L^−1^ are commonly considered deficient or inadequate, whereas values between 50 and 75 nmol·L^−1^ are often considered insufficient [96]. Athlete-focused literature has used higher classification boundaries, including approximately 75–80 nmol·L^−1^, but definitions are heterogeneous and should not be interpreted as validated universal performance or musculoskeletal targets [95]. The optimal athlete-specific 25(OH)D concentration remains uncertain and is likely to depend on outcome and context.

The central interpretive risk is therefore twofold: under-recognizing suboptimal vitamin D status in at-risk athletes, and assuming that higher 25(OH)D is always better. The practical goal is to identify deficiency, insufficiency, or potentially suboptimal status that may compromise musculoskeletal health, immune function, or bone adaptation, while avoiding indiscriminate supplementation or unnecessary pursuit of high concentrations [95,96]. Conversely, in athletes with adequate 25(OH)D, persistent fatigue, injury, or poor performance should not be automatically attributed to vitamin D; other explanations such as low energy availability, inadequate carbohydrate intake, iron deficiency, sleep disruption, infection, or excessive load may be more plausible [17,25].

### 8.2. Calcium, Phosphate, PTH, and Bone Turnover

Calcium and phosphate are essential for hydroxyapatite formation, muscle contraction, nerve conduction, intracellular signaling, ATP metabolism, and acid–base regulation. PTH responds to changes in ionized calcium and vitamin D status by increasing renal calcium reabsorption, phosphate excretion, and renal 1α-hydroxylase activity, while also influencing bone remodeling when chronically elevated [96]. In athletes, calcium–phosphate–PTH interpretation is particularly relevant when 25(OH)D is low, bone stress injury is suspected, dietary restriction is present, or REDs risk is high [17,97].

Bone turnover markers provide a dynamic view of skeletal remodeling. P1NP reflects type I collagen formation and is commonly used as a bone formation marker, whereas CTX-I reflects type I collagen degradation and is used as a bone resorption marker. OC, BALP, sclerostin, and related markers add further information on osteoblast activity, mineralization, and osteocyte signaling [98]. Stunes et al. [98] observed that acute strength and endurance exercise altered bone turnover and related markers in a sex- and age-dependent manner, with responses generally returning toward baseline within 24 h. This illustrates why bone biomarkers require careful timing relative to exercise.

The mechanobiology of bone differs from muscle. A single bout of loading may transiently alter P1NP, CTX-I, OC, or sclerostin, but chronic skeletal adaptation depends on repeated loading cycles, strain magnitude, strain rate, recovery, energy availability, endocrine status, and nutrient sufficiency [98]. Consequently, an isolated P1NP or CTX-I value after a recent training session should not be interpreted as evidence of long-term bone gain or bone loss. A more defensible interpretation is whether the athlete’s loading, nutrition, and endocrine environment appear compatible with healthy remodeling [98].

Calcium and vitamin D supplementation may reduce stress fracture incidence in certain high-risk populations. Lappe et al. [99] observed that calcium and vitamin D supplementation decreased stress fracture incidence in female Navy recruits, a physically active population exposed to intense training. However, extrapolation to all athletes should be cautious because baseline status, training load, diet, sex, age, and injury risk differ across populations [97,99]. Supplementation decisions should therefore be based on deficiency risk, dietary assessment, biomarkers, and clinical context rather than a blanket strategy.

### 8.3. Stress Fracture Risk and Energy Availability

Bone stress injuries occur when repetitive skeletal loading exceeds the capacity for microdamage repair and remodeling. Biomarkers alone cannot diagnose bone stress injury, but they may help identify biological conditions that increase risk, such as low 25(OH)D, low calcium intake, low energy availability, menstrual dysfunction, low sex hormones, suppressed bone formation, or elevated bone resorption [17,97]. The key issue is that mechanical load and metabolic context interact: the same training load may be tolerated in an adequately fueled athlete but become injurious in an athlete with impaired remodeling capacity [17,100].

Hutson et al. [100] reviewed the effects of low energy availability on bone health in endurance athletes and emphasized that LEA may reduce bone mineral density, alter bone geometry, and impair bone remodeling. Mechanistically, LEA can suppress hypothalamic–pituitary–gonadal function, reduce insulin-like growth factor-1, alter thyroid hormones, increase cortisol, and impair bone formation, thereby weakening the skeletal response to repetitive loading [17,100].

The Female Athlete Triad literature remains highly relevant within the broader REDs framework. De Souza et al. [101] described the interrelationship between low energy availability, menstrual dysfunction, and low bone mineral density, and proposed risk stratification for treatment and return-to-play decisions. In biomarker terms, this means that 25(OH)D, calcium, PTH, P1NP, CTX-I, ferritin, reproductive hormones, thyroid markers, and inflammatory markers may provide useful context, but none should be interpreted independently from menstrual history, dietary intake, training load, injury history, and bone imaging when clinically indicated [101].

For endurance and aesthetic sport athletes, the most concerning pattern is convergence rather than a single abnormal marker: low or borderline 25(OH)D, low calcium intake, low ferritin or iron availability, menstrual dysfunction or low testosterone, weight loss, recurrent injury, high training volume, poor recovery, and declining performance [17,100]. In such cases, bone turnover markers may support the interpretation, but clinical assessment and imaging remain necessary when bone stress injury is suspected [97,101].

### 8.4. When Supplementation Should and Should Not Be Inferred from Biomarkers

Biomarkers can identify deficiency, insufficiency, or physiological risk states, but they should not automatically justify supplementation without considering dose, baseline status, toxicity risk, and the likelihood that the biomarker is causally related to the athlete’s problem [95,96]. Vitamin D provides a clear example. Correcting deficiency is biologically plausible and clinically important, but supplementing already sufficient athletes is less likely to improve performance and may create false reassurance if other causes of fatigue, injury, or poor adaptation are ignored [95,96,97].

Calcium and vitamin D should be interpreted as part of a bone health system rather than as isolated supplements. Lappe et al. [99] provided evidence of stress fracture reduction in female recruits, but this does not mean supplementation alone can compensate for excessive training load, low energy availability, menstrual dysfunction, poor sleep, inadequate total diet, or premature return to impact loading. Supplementation may support bone remodeling only when the broader training–nutrition–recovery environment is adequate [99,100,101].

Bone turnover markers should also not be used simplistically to prescribe supplements. A high CTX-I may reflect fasting, circadian timing, recent exercise, low energy availability, or bone resorption, whereas P1NP may reflect bone formation but changes slowly and is influenced by growth, training history, and recovery [98]. Therefore, the most appropriate use of these markers is longitudinal interpretation in athletes at risk, not immediate decisions from one sample [25,98].

A practical panel for suspected bone–muscle health risk may include 25(OH)D, calcium, phosphate, PTH, creatinine, ferritin and iron status, CRP, thyroid markers, reproductive hormones when clinically indicated, and bone turnover markers such as P1NP and CTX-I when available [17,97]. The decision to supplement or medically evaluate should depend on symptoms, diet, menstrual or endocrine status, training load, fracture history, and imaging findings rather than biomarkers alone [97,101].

## 9. Inflammatory and Immunometabolic Biomarkers

**Figure 5:**
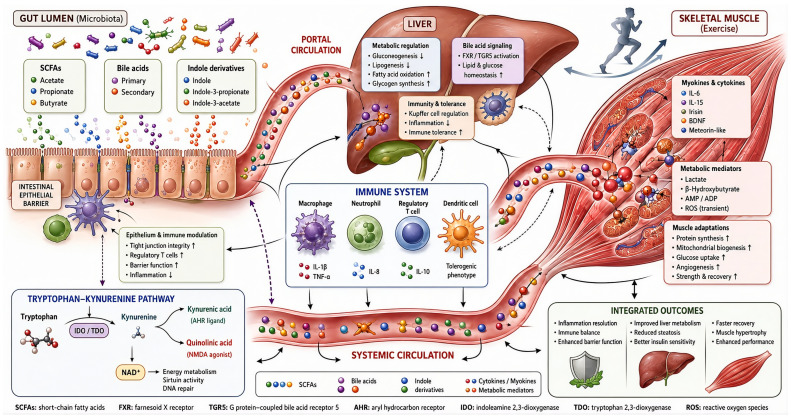
Immunometabolic network linking exercise, inflammation, and microbial metabolites. Exercise and gut-derived metabolites interact through a gut–liver–immune–muscle axis linking microbial products, intestinal barrier function, hepatic metabolism, immune regulation, tryptophan–kynurenine metabolism, and muscle-derived cytokines and metabolic mediators. Solid black arrows illustrate direct transport routes via portal and systemic circulation, direct metabolic conversions, and the secretion of biological mediators. Dashed black arrows indicate indirect signaling, cross-talk, and modulatory feedback between different organ systems (e.g., gut–liver and liver–muscle interactions). Dotted purple arrows specifically highlight the influence of gut and immune modulation on the tryptophan–kynurenine pathway and systemic circulation. Thick double-headed black arrow connects the entire systemic immunometabolic network to the resulting integrated clinical and physiological outcomes. Together, these pathways contribute to integrated outcomes relevant to inflammation resolution, metabolic health, recovery, and exercise adaptation. Abbreviations: AHR, aryl hydrocarbon receptor; AMP/ADP, adenosine monophosphate/adenosine diphosphate; BDNF, brain-derived neurotrophic factor; FXR, farnesoid X receptor; IDO, indoleamine 2,3-dioxygenase; IL-1β, interleukin-1 beta; IL-6, interleukin-6; IL-8, interleukin-8; IL-10, interleukin-10; IL-15, interleukin-15; NAD^+^, nicotinamide adenine dinucleotide; NMDA, N-methyl-D-aspartate; ROS, reactive oxygen species; SCFAs, short-chain fatty acids; TDO, tryptophan 2,3-dioxygenase; TGR5, G protein-coupled bile acid receptor 5; TNF-α, tumor necrosis factor alpha.

Inflammatory and immunometabolic biomarkers are central to athlete monitoring because exercise affects immune cell trafficking, cytokine release, acute-phase proteins, muscle repair, substrate metabolism, gut barrier function, and host–microbiome signaling (Figure 5). C-reactive protein (CRP), high-sensitivity CRP (hs-CRP), interleukin-6 (IL-6), interleukin-1 beta (IL-1β), interleukin-8 (IL-8), interleukin-10 (IL-10), tumor necrosis factor-α (TNF-α), leukocyte subsets, kynurenine pathway metabolites, short-chain fatty acids (SCFAs), bile acids, indoles, and selected lipid mediators may all change with training, illness, recovery, and nutrition [102,103,104]. However, most inflammatory markers are nonspecific; they indicate immune–metabolic activation rather than a single diagnosis [10,102].

The interpretive problem is that inflammation can be adaptive or maladaptive. Acute post-exercise inflammation contributes to tissue remodeling, substrate mobilization, and immune surveillance, while chronic or excessive inflammation may signal infection, injury, poor recovery, low energy availability, excessive load, or cardiometabolic stress [10,102]. Therefore, inflammatory biomarkers should be interpreted according to timing, magnitude, symptoms, recent muscle damage, sleep, travel, nutrition, and performance rather than as independent “readiness” markers [4,16].

### 9.1. C-Reactive Protein and hs-CRP

CRP is an acute-phase protein produced mainly by the liver in response to IL-6 and other inflammatory signals. It is useful as a broad marker of systemic inflammation, but it is not specific to infection, injury, muscle damage, cardiometabolic risk, or training stress [102]. Kasapis and Thompson [102] reviewed the effects of physical activity on CRP and inflammatory markers, showing that acute and chronic exercise can have different associations with inflammation. For athletes, this means that a transient CRP increase after competition should not be interpreted in the same way as persistent elevation during fatigue, illness, or unexplained performance decline [102].

Exercise training may reduce basal CRP in many populations, but this effect depends on baseline inflammatory status, adiposity, training dose, and health condition. Fedewa et al. [103] showed in a systematic review and meta-analysis that exercise training can reduce CRP, particularly in individuals with higher baseline values. In athletes, however, low-grade CRP elevations may also reflect recent mechanical trauma, high competition density, respiratory infection, or tissue remodeling rather than chronic cardiometabolic inflammation [10,103].

hs-CRP assays improve sensitivity at low concentrations and may be useful when interpreted longitudinally and with symptom context. A mild hs-CRP increase after an intense match, downhill race, or heavy eccentric session may reflect tissue stress; a marked elevation with fever, sore throat, malaise, reduced performance, or leukocytosis should prompt concern for infection or other pathology [10,13]. CRP is also useful for interpreting iron status, because inflammation can raise ferritin and hepcidin while reducing serum iron and TSAT, creating a pattern of functional iron restriction if ferritin is interpreted alone [84,88].

### 9.2. Acute Exercise Inflammation Versus Infection or Injury

Acute exercise triggers a coordinated immune response involving leukocyte mobilization, cytokine release, acute-phase signaling, and tissue repair. Docherty et al. [104] reviewed cytokine responses to exercise and emphasized that IL-6, IL-10, TNF-α, and other cytokines vary according to intensity, duration, muscle damage, training status, and sampling time. A post-exercise cytokine profile is therefore not inherently pathological; it may represent normal immune–metabolic signaling [104].

The distinction between exercise inflammation and infection depends on convergence of signals. Exercise-related inflammation usually follows a plausible training or competition stimulus, has expected kinetics, and resolves with recovery. Infection is more likely when inflammatory markers are accompanied by fever, chills, sore throat, cough, gastrointestinal symptoms, persistent malaise, abnormal leukocyte patterns, or disproportionate performance decline [13,102]. Injury-related inflammation becomes more plausible when localized pain, swelling, loss of function, or imaging findings accompany biomarker changes [10].

Timing is critical. A sample collected within hours of a marathon, rugby match, heavy eccentric session, or ultra-endurance event may show leukocytosis, elevated CRP, altered cytokines, increased CK, and broad metabolic perturbation [10,104]. Interpreting that sample as “poor health” without considering the exercise stimulus would be inappropriate. Conversely, persistent inflammatory elevation outside the expected recovery window should prompt review of illness, injury, sleep, nutrition, load, and psychological stress [4,13].

Inflammation also interacts directly with metabolism. IL-6 and other cytokines influence hepatic glucose output, lipolysis, insulin sensitivity, hepcidin, appetite, immune cell–substrate use, and muscle repair [105]. At the same time, the inflammatory response to exercise often occurs alongside changes in lactate, amino acids, acylcarnitines, fatty acids, oxylipins, and kynurenine pathway metabolites, reflecting coordinated regulation of immune signaling, substrate availability, redox balance, and tissue remodeling [19,35]. This overlap helps explain why inflammatory markers should be interpreted together with metabolic, nutritional, and recovery context rather than as isolated indicators of illness or maladaptation.

### 9.3. Cytokines, Myokines, and Exercise-Induced Immune Signaling

IL-6 is the archetypal exercise-responsive myokine. Pedersen and Fischer [105] proposed that muscle-derived IL-6 acts as an exercise factor with metabolic and anti-inflammatory properties, challenging the assumption that IL-6 is always harmful. During exercise, IL-6 can increase markedly, especially with prolonged duration, low glycogen availability, and large active muscle mass, and may stimulate hepatic glucose production, lipolysis, fat oxidation, and anti-inflammatory cytokine responses [105].

Starkie et al. [106] observed that exercise and IL-6 infusion inhibited endotoxin-induced TNF-α production in humans, supporting the concept that exercise-induced IL-6 can participate in anti-inflammatory signaling. This illustrates why cytokines cannot be interpreted by disease-based assumptions alone: IL-6 elevation during sepsis, obesity, chronic inflammation, and exercise may differ in source, kinetics, receptor context, and downstream effects [105,106].

Gleeson et al. [107] reviewed the anti-inflammatory effects of exercise and described mechanisms involving reduced visceral adiposity, myokine-mediated cytokine regulation, immune cell phenotype shifts, and changes in Toll-like receptor signaling. Nieman and Wentz [108] further emphasized that habitual moderate exercise supports immune defense, while prolonged intense training and competition may transiently increase illness susceptibility in some athletes. Cytokine interpretation therefore needs to account for both the acute bout and the athlete’s chronic training–recovery balance [107,108].

From a metabolomic perspective, cytokines are regulators of substrate flow and immune cell metabolism. Exercise-induced cytokines can influence glucose, fatty acid, amino acid, and iron metabolism; conversely, nutrient availability and metabolic stress can shape cytokine responses [105,107]. For example, low glycogen availability can amplify IL-6 release during exercise, while inflammation can alter tryptophan metabolism through indoleamine 2,3-dioxygenase activation and the kynurenine pathway [105,109].

### 9.4. Tryptophan–Kynurenine Pathway, Fatigue, and Mental Health

The tryptophan–kynurenine pathway is a promising immunometabolic axis linking exercise, inflammation, brain function, fatigue, and metabolic health. Tryptophan can be metabolized through serotonin, melatonin, indole, and kynurenine pathways. Under inflammatory conditions, indoleamine 2,3-dioxygenase and tryptophan 2,3-dioxygenase activity can increase conversion of tryptophan to kynurenine, which can be further metabolized into kynurenic acid, quinolinic acid, picolinic acid, xanthurenic acid, and NAD^+^-related metabolites [109].

Agudelo et al. [109] observed that skeletal muscle peroxisome proliferator-activated receptor gamma coactivator 1-alpha-1 (PGC-1α1) modulates kynurenine metabolism and may protect against stress-induced depression by increasing kynurenine aminotransferase expression and conversion of kynurenine to kynurenic acid. This mechanism provides a biologically plausible link between endurance training, skeletal muscle adaptation, and mental health, and reframes skeletal muscle as an organ capable of redirecting circulating metabolites with neuroactive potential [109].

In later work, Agudelo et al. [110] reported that PGC-1α1 activates the malate–aspartate shuttle in skeletal muscle, supported by kynurenine catabolism, as part of endurance exercise adaptation. This connects amino acid metabolism, NADH/NAD^+^ redox transfer, mitochondrial respiration, and exercise adaptation [110]. In athletes, kynurenine pathway metabolites may eventually help characterize inflammatory load, stress resilience, central fatigue, or adaptation, but they are not yet validated routine monitoring markers [109,110].

Interpretation of kynurenine metabolites requires caution. Kynurenine, kynurenic acid, quinolinic acid, and related ratios can be influenced by inflammation, infection, psychological stress, diet, gut microbiota, sleep, sex hormones, medications, and sampling matrix [109,110]. Their strongest current value is mechanistic: they highlight how exercise-induced muscle adaptations may shape systemic immunometabolic signaling [109,110].

### 9.5. Microbiome-Derived Metabolites: SCFAs, Bile Acids, and Indoles

The gut microbiome is increasingly recognized as a potential contributor to exercise metabolism, immune regulation, gastrointestinal function, and host–microbe signaling. Exercise may influence microbial diversity, barrier function, immune signaling, and microbial metabolite production, but the translation of these findings into athlete monitoring remains limited [111,112]. In athletes, microbiome-derived metabolites should therefore be interpreted primarily as mechanistic and exploratory signals rather than as practical readiness, recovery, or performance biomarkers.

SCFAs, particularly acetate, propionate, and butyrate, are microbial metabolites derived from fermentation of dietary fibers and resistant starches. They can influence gut barrier function, immune regulation, glucose metabolism, lipid metabolism, and host energy signaling through G-protein-coupled receptors and histone deacetylase inhibition [113]. Bile acids represent another gut–liver–microbiome metabolite class, linking lipid absorption, farnesoid X receptor signaling, Takeda G protein-coupled receptor 5 signaling, glucose metabolism, energy expenditure, and inflammation [114]. Indole derivatives connect microbial tryptophan metabolism with intestinal barrier function, aryl hydrocarbon receptor signaling, immune regulation, and host metabolism [115].

Despite this biological plausibility, SCFAs, bile acids, and indoles are not yet validated biomarkers for routine athlete monitoring. Existing exercise–microbiome studies are often observational, heterogeneous, and highly vulnerable to confounding by diet, fiber intake, protein intake, supplements, antibiotics, travel, gastrointestinal symptoms, body composition, training load, sport type, and sampling method [116,117,118]. Studies in athletes also show that microbiome composition and function may differ from sedentary controls, but causal interpretation is difficult because elite sport involves many concurrent exposures, including diet, body size, training volume, recovery behaviors, and environmental factors [116].

The current value of microbiome-derived metabolites is therefore conceptual rather than prescriptive. They may help explain how diet, training, gut barrier function, inflammation, substrate availability, and recovery interact, but they should not be used to guide training load, supplementation, recovery status, or clinical decisions in isolation. For now, their most defensible role is in research models that combine standardized dietary assessment, longitudinal sampling, gastrointestinal symptoms, inflammatory markers, metabolomics, and performance outcomes. 

## 10. Endocrine Biomarkers of Stress, Recovery, and Energy Availability

Endocrine biomarkers are attractive in athlete monitoring because hormones integrate training load, energy availability, sleep, psychological stress, substrate mobilization, reproductive function, tissue remodeling, and recovery. Cortisol, testosterone, reproductive and thyroid hormones, insulin, leptin, growth hormone (GH), insulin-like growth factor-1 (IGF-1), catecholamines, and sex hormone-binding globulin (SHBG) may respond to different dimensions of exercise and nutritional stress [15,119]. However, endocrine biomarkers are particularly vulnerable to overinterpretation because they are pulsatile, circadian, matrix-dependent, sex- and energy-sensitive, and influenced by sleep, travel, illness, psychological stress, reproductive context, recent exercise, training status, and exercise mode [120,121]. They therefore generally reflect total physiological context rather than training load alone and should be interpreted longitudinally, under standardized sampling conditions, and in relation to a clear clinical or performance question [4,25].

From a metabolomics-informed perspective, endocrine changes may be situated within broader signatures of substrate availability, lipid mobilization, amino acid turnover, ketogenesis, inflammatory signaling, and energy conservation. This is relevant because similar hormonal changes may arise from different combinations of training exposure, low energy availability, sleep disruption, illness, or psychological stress [17,119]. Pathway-level metabolic profiles may eventually help distinguish isolated hormonal variation from coordinated endocrine–metabolic suppression, but their incremental value over established clinical, nutritional, and functional assessment remains unproven [20,21,22]. Accordingly, metabolomics should be viewed as a complementary contextual layer rather than a replacement for established endocrine assessment.

The main endocrine biomarkers discussed in this section, their physiological interpretation, and major sources of confounding are summarized in Table 4.

### 10.1. Cortisol: Circadian Rhythm and Total Stress Load

Cortisol is the primary glucocorticoid of the hypothalamic–pituitary–adrenal (HPA) axis and plays a central role in glucose mobilization, lipolysis, protein turnover, vascular tone, immune modulation, and stress adaptation. In exercise, cortisol may increase with prolonged endurance work, high-intensity exercise, low carbohydrate availability, psychological stress, sleep disruption, illness, travel, and congested competition schedules [119,121]. It should therefore be considered a broad marker of total stress load rather than a specific marker of training load.

The main interpretive challenges are circadian variation and sample matrix. Cortisol typically peaks in the early morning and declines across the day, while sleep timing, light exposure, food intake, caffeine, psychological stress, and recent exercise may alter this pattern [119,122]. Serum, plasma, saliva, and urine also reflect different fractions or integrations of cortisol biology. Salivary cortisol is attractive in sport because it is non-invasive and may approximate free cortisol, but it remains sensitive to collection timing, oral contamination, flow rate, food intake, assay type, and protocol adherence [122]. Consequently, values obtained at different times or from different matrices should not be treated as interchangeable.

A single high cortisol value should rarely drive a training decision. Basal endocrine markers have generally shown limited value as standalone indicators of overtraining-related states, whereas altered patterns or dynamic responses may be more informative in selected contexts [119]. Cortisol becomes more actionable when a sustained change converges with poor sleep, high perceived stress, mood disturbance, elevated RPE, illness symptoms, impaired performance, or low energy availability [15,17].

### 10.2. Testosterone: Anabolic Status, Recovery, and Confounding

Testosterone is often interpreted as an anabolic hormone related to muscle protein synthesis, erythropoiesis, neuromuscular function, mood, libido, recovery, and training adaptation. In men, circulating testosterone reflects hypothalamic–pituitary–gonadal (HPG) axis function, testicular production, SHBG binding, energy status, sleep, illness, and training history [121,123,124]. In women, testosterone is present at lower concentrations and is influenced by ovarian, adrenal, and peripheral metabolism, making assay sensitivity, menstrual-cycle context, hormonal contraceptive use, and clinical interpretation particularly important [125].

Resistance exercise can acutely increase testosterone depending on volume, intensity, muscle mass recruited, rest intervals, training status, and nutritional state [123]. However, acute post-exercise increases do not necessarily translate directly into hypertrophy or performance improvement, and chronic adaptation cannot be inferred from single acute hormonal spikes [123]. Therefore, testosterone should not be treated as a simple anabolic “score”; it is one part of a broader endocrine–metabolic environment that includes energy availability, protein intake, sleep, training load, and recovery [120,123,124].

Hackney [124] described the “exercise-hypogonadal male condition” as a pattern in which men engaged in high-volume endurance training may present with chronically reduced resting testosterone and altered HPG-axis regulation. This does not automatically imply disease in every endurance-trained athlete, but it highlights that chronic training exposure can shift endocrine set-points [124]. In practice, low testosterone becomes more concerning when accompanied by low libido, mood changes, fatigue, poor recovery, bone stress injury, low energy availability, reduced performance, or other endocrine abnormalities [17,124,125,126,127,128,129].

Testosterone measurement also depends strongly on SHBG and assay quality. Total testosterone may be misleading when SHBG is altered by energy availability, thyroid status, liver function, illness, medications, or sex hormones. Free testosterone or calculated free testosterone may provide additional context, but these estimates also depend on assay validity and model assumptions [122,125]. Testosterone should therefore be interpreted with timing, symptoms, sex, age, training phase, sleep, energy availability, menstrual or reproductive context, and SHBG whenever possible [122,124,125].

### 10.3. Testosterone-to-Cortisol Ratio: Use with Caution

The testosterone-to-cortisol ratio (T:C) has historically been used as an index of anabolic–catabolic balance, but this interpretation is overly simplified because testosterone and cortisol arise from different endocrine axes, have distinct circadian patterns, respond to different stimuli, and present different analytical limitations [119,120,122]. A reduced T:C ratio may accompany short-term physiological strain, but it does not diagnose overtraining syndrome, predict injury, or determine readiness; conversely, a stable ratio does not exclude fatigue, low energy availability, illness, or impaired recovery [4,15,120].

The ratio is also mathematically fragile because a change may result from increased cortisol, decreased testosterone, both, or analytical noise. Components should therefore be examined separately before the ratio is interpreted [120,122]. In practice, T:C may be supportive only when sampling is standardized, individual baselines exist, and sustained changes converge with performance, symptoms, sleep, training load, and neuromuscular or perceptual markers [25,122,130]. It should not be used as a standalone traffic-light or diagnostic biomarker [15].

### 10.4. Thyroid Axis, Leptin, Insulin, and Metabolic Suppression

Low energy availability can alter multiple endocrine axes beyond testosterone and cortisol. Thyroid hormones, leptin, insulin, GH, IGF-1, LH pulsatility, estradiol, progesterone, and reproductive function may all shift as part of energy-conservation physiology [17,126,127,128,129]. These changes should often be understood as metabolic adaptations to insufficient energy availability rather than isolated endocrine abnormalities [119,128].

Loucks et al. [126] observed that low energy availability, rather than the stress of exercise itself, altered LH pulsatility in exercising women. Later, Loucks and Thuma [127] identified a threshold-like disruption of LH pulsatility below approximately 30 kcal·kg^−1^ fat-free mass·day^−1^, with associated changes in metabolic hormones. These findings are foundational because they show that reproductive-axis suppression can arise from inadequate energy availability even in the absence of overt eating disorder pathology [126,127].

Areta et al. [128] emphasized that low energy availability is a physiological state with definitional, mechanistic, and evidentiary complexity, not merely a dietary behavior. Loucks et al. [129] also framed energy availability as a central concept in athletes, linking exercise energy expenditure with the residual energy available for physiological functions. In biomarker terms, low triiodothyronine (T3), low leptin, altered insulin, reduced IGF-1, menstrual dysfunction, low testosterone, or impaired bone turnover may represent a coordinated energy-conservation response [17,128,129].

Insulin and leptin are particularly relevant because they signal short- and long-term energy status. Low insulin may reflect low carbohydrate availability, prolonged exercise, or energy deficit, while low leptin may reflect low fat mass, acute energy restriction, or chronic low energy availability [126,129]. These markers are not routine athlete-monitoring tools in many settings, but they can help contextualize unexplained fatigue, menstrual dysfunction, recurrent bone stress injury, or endocrine suppression when used clinically [17,129].

### 10.5. Endocrine Disruption in Overreaching, Overtraining, and REDs

Functional overreaching, non-functional overreaching, overtraining syndrome, and REDs may overlap clinically but are not equivalent. Overreaching and overtraining are primarily load–recovery maladaptation constructs, whereas REDs centers on low energy availability and its multisystem consequences [15,17]. Endocrine biomarkers may be altered across these states, but no single hormone, ratio, or basal endocrine profile reliably distinguishes them [15,119]. Persistent performance decrement, fatigue, and exclusion of competing medical, nutritional, psychological, or infectious explanations remain central to overtraining-syndrome assessment [15].

Accordingly, low testosterone, high cortisol, reduced T:C, altered thyroid markers, suppressed leptin, or blunted hormonal responses may support concern only when they converge with symptoms and longitudinal functional impairment [15,119,120]. In REDs, coordinated changes in reproductive hormones, thyroid signaling, leptin, IGF-1, and bone metabolism may accompany low energy availability before severe clinical consequences become evident [17,128]. Interpretation should integrate dietary assessment, training load, psychological factors, symptoms, bone health, and performance trajectory rather than attributing an endocrine pattern to training stress alone [15,17,125,129].

Sex and reproductive context are essential. In men, persistent low testosterone or low libido may provide relevant clues; in women, menstrual disturbance, low estradiol, altered luteal function, or amenorrhea may indicate reproductive-axis suppression [17,125,126]. Menstrual-cycle phase, hormonal contraceptive use, and reproductive status should therefore be considered when interpreting estradiol, progesterone, LH, FSH, cortisol, and related metabolic responses [125].

## 11. Systems-Level Metabolomics and Integrative Athlete Monitoring

Section 4, Section 5, Section 6, Section 7, Section 8, Section 9 and Section 10 examined major physiological domains using routine biomarkers as accessible but biologically nonspecific signals, with metabolomic evidence incorporated where relevant. This section shifts the level of analysis from domain-based interpretation toward systems-level metabolic patterns. Emerging metabolomics provides a way to move from single-analyte monitoring toward coordinated interpretation of exercise biology across glycolysis, glycogenolysis, β-oxidation, ketogenesis, amino acid turnover, purine degradation, redox balance, bile acid metabolism, lipid remodeling, mitochondrial substrate flux, immune-metabolic signaling, and interorgan communication [18,20,35]. The challenge is not only to detect metabolites, but to interpret them in relation to exercise mode, intensity, duration, recovery timing, nutrition, sex, training status, and sport context [21,48].

A metabolomics-informed approach does not replace routine biomarkers; it contextualizes them. Lactate, glucose, urea, CK, creatinine, ferritin, CRP, cortisol, and testosterone remain useful because they are accessible and interpretable in applied settings [3,4]. However, metabolomics can reveal whether these routine changes occur within broader signatures of substrate depletion, mitochondrial overload, oxidative stress, purine catabolism, inflammatory lipid signaling, gut-derived metabolites, or endocrine–metabolic suppression [18,19].

### 11.1. Acute Exercise Metabolomics

Acute exercise metabolomics has shown that a single exercise bout can generate rapid, coordinated, and time-dependent changes in circulating metabolites. Lewis et al. [18] demonstrated that exercise produces plasma signatures involving glycogenolysis, lipolysis, TCA-cycle intermediates, amino acids, and metabolites associated with insulin sensitivity and mitochondrial metabolism. Contrepois et al. [19] later observed complex multi-omic changes after acute exercise, including metabolomic, proteomic, inflammatory, and cardiovascular pathway responses.

Hawley et al. [131] described exercise adaptation as an integrative biological phenomenon involving skeletal muscle, liver, adipose tissue, vasculature, endocrine organs, immune cells, and nervous system regulation. This is central to metabolomics because circulating metabolites are not produced by a single tissue. Lactate may arise from working muscle, red blood cells, and other glycolytic tissues; ketone bodies largely reflect hepatic metabolism; bile acids reflect gut–liver signaling; acylcarnitines reflect mitochondrial substrate handling; and purines reflect ATP turnover and nucleotide degradation [35,48,131].

The acute metabolomic response is strongly time-dependent. Sampling immediately after exercise captures different biology from samples collected 1 h, 3 h, 24 h, or 48 h later [19,48]. Early changes may reflect lactate, pyruvate, succinate, catecholamine-sensitive lipolysis, purine degradation, and acid–base stress; later changes may reflect inflammation, repair, lipid remodeling, amino acid turnover, glycogen restoration, renal excretion, and endocrine recovery [19,48].

Exercise mode also matters. Morville et al. [24] observed both overlapping and distinct plasma metabolome responses after resistance and endurance exercise. This supports the idea that metabolomics may help characterize the biological fingerprint of different training stimuli. A resistance session may emphasize amino acid turnover, mechanical stress, and repair-related pathways, whereas endurance exercise may emphasize lipid mobilization, TCA intermediates, ketone bodies, acylcarnitines, and prolonged substrate flux [24,35].

### 11.2. Chronic Training Adaptations and Metabotypes

Chronic training modifies both the resting metabolome and the response to acute exercise. These adaptations may include improved mitochondrial oxidative capacity, altered lipid handling, enhanced lactate clearance, greater metabolic flexibility, modified amino acid turnover, reduced basal inflammation, and more efficient recovery kinetics [20,21]. However, the direction and magnitude of change depend on training modality, dose, baseline fitness, nutrition, sex, age, and health status [20,21].

The Molecular Transducers of Physical Activity Consortium (MoTrPAC) was established to map the molecular responses to exercise across tissues and time. Sanford et al. [132] described the consortium’s goal of generating an integrative multi-omic map of dynamic exercise responses, including human clinical exercise studies with endurance and resistance exercise, blood, skeletal muscle, and adipose tissue sampling. This type of initiative is important because athlete metabolomics cannot be fully interpreted from blood alone; plasma is a useful window, but tissue-specific responses in skeletal muscle, adipose tissue, liver, heart, and immune cells may differ substantially [131,132].

For the purposes of athlete interpretation, human data should be prioritized whenever possible. Human multi-omic exercise studies show that acute and chronic exercise responses are tissue-, time-, sex-, and modality-dependent, and that circulating metabolites represent only one layer of a broader molecular response involving skeletal muscle, adipose tissue, immune signaling, cardiovascular regulation, and endocrine–metabolic adaptation [19,24,131,132,133]. This supports the use of metabolomics as a contextual layer, but also cautions against inferring individual adaptation from a single circulating metabolite panel.

Sex and hormonal context are also important for metabolomics-informed monitoring. Pataky et al. [133] showed that biological sex and sex hormones are associated with molecular signatures of skeletal muscle at rest and in response to distinct exercise training modes in humans. This reinforces that metabolomic and multi-omic interpretation should not assume identical baseline profiles, responses, or recovery kinetics in male and female athletes. Sex, menstrual status, hormonal contraceptive use, reproductive function, age, and training modality should therefore be integrated into future metabolomic study designs and, when feasible, into applied interpretation [125,133].

### 11.3. Metabolomic Signatures of Training Response

One of the most promising applications of metabolomics is the identification of baseline metabotypes or acute-response signatures that may help characterize heterogeneity in training adaptation. In principle, metabolites related to mitochondrial function, lipid oxidation, amino acid turnover, purine degradation, redox regulation, and inflammatory signaling could help describe biological differences between athletes exposed to the same training stimulus [20,21]. However, this remains an emerging area and requires careful validation before individual training decisions are made [22].

Metabolomic predictors should be interpreted probabilistically, not deterministically. A baseline profile rich in acylcarnitines, branched-chain amino acids, inflammatory lipids, or altered bile acids may indicate metabolic phenotype, but it does not by itself determine training response [20,35]. Similarly, an acute post-exercise metabolite signature may reflect recent diet, glycogen availability, sleep, sampling time, environmental conditions, or assay platform rather than stable biological responsiveness [48].

A defensible pathway is to treat metabolomics as hypothesis-generating until repeated evidence shows that a metabolite panel predicts a clinically or performance-relevant outcome beyond simpler markers. For example, a metabolomic model should be tested against practical comparators such as training history, performance tests, RPE, sleep, lactate thresholds, CK, CRP, ferritin, and energy availability indicators [4,16]. The relevant question is not whether metabolomics can detect biological variation, but whether it improves decisions beyond existing tools [22].

### 11.4. Exerkines and Interorgan Metabolic Communication

The concept of exerkines has expanded the biological interpretation of exercise-induced circulating molecules. Exerkines include peptides, proteins, metabolites, lipids, nucleic acids, extracellular vesicles, and other signaling moieties released in response to acute or chronic exercise that mediate local or systemic effects [134,135]. Several exerkines are small metabolites or lipid mediators, making this framework useful for interpreting exercise as a coordinated interorgan signaling event rather than a purely muscular stimulus [135,136].

Whitham et al. [134] observed that exercise increases circulating extracellular vesicles containing proteins with potential roles in tissue crosstalk, suggesting that exercise-induced communication is not limited to freely circulating metabolites or classical hormones. Chow et al. [135] defined exerkines as signaling moieties released in response to exercise and discussed their roles in health, resilience, and disease. In athletes, exerkines provide a framework for interpreting exercise as a systemic molecular intervention rather than a purely muscular event [135,136].

Myokines and metabolite-like exerkines may influence liver, adipose tissue, pancreas, brain, bone, immune cells, and vasculature. Laurens et al. [136] reviewed exercise-released myokines involved in energy metabolism, including effects on skeletal muscle, adipose tissue, liver, and pancreas. This interorgan perspective helps explain why exercise changes metabolites such as lactate, alanine, glutamine, β-hydroxybutyrate, β-aminoisobutyric acid (BAIBA), Lac-Phe, kynurenine derivatives, SCFAs, bile acids, and acylcarnitines in ways that cannot be assigned to one tissue alone [35,131,132,136].

Li et al. [137] identified Lac-Phe as an exercise-inducible metabolite linked to appetite suppression and obesity protection in preclinical models. Roberts et al. [138] identified BAIBA as a PGC-1α-related small molecule associated with browning of white adipose tissue and hepatic β-oxidation. These examples illustrate how exercise-induced metabolites may act as signals, not merely by-products [137,138]. Nevertheless, neither BAIBA nor Lac-Phe should yet be used as routine athlete-monitoring biomarkers without stronger sport-specific validation [22,137].

### 11.5. Integrating Routine Biomarkers, Omics, and Wearables

The future of athlete monitoring is likely to be integrative rather than omics-only. Wearables and field tests provide high-frequency information on external load, heart rate, heart rate variability (HRV), sleep, body temperature, global positioning system (GPS)-derived movement, accelerometry, power output, and recovery patterns, whereas blood biomarkers and metabolomics provide lower-frequency but deeper biological information [5,34]. Combining these layers may help distinguish similar symptoms with different underlying biology—for example, fatigue due to glycogen depletion, iron deficiency, infection, muscle damage, heat strain, or low energy availability [4,17].

Sato et al. [139] developed an atlas of exercise metabolism showing time-dependent signatures of metabolic homeostasis across tissues and time-of-day contexts. This is relevant because metabolomic interpretation depends on circadian timing, tissue specificity, and sampling schedule [139]. In athletes, the same metabolite panel may have different meaning in the morning fasted state, after evening training, during a congested competition week, or after travel across time zones [139].

Integration should proceed from the practical question. If the question is intensity prescription, lactate, ventilatory thresholds, critical power, and performance measures may be primary. If the question is recovery after muscle-damaging exercise, CK, myoglobin, soreness, jump performance, acylcarnitines, purines, and inflammatory lipid mediators may be relevant. If the question is low energy availability, endocrine markers, body-mass trend, dietary assessment, urea, amino acids, thyroid markers, leptin, reproductive hormones, and bone turnover may be considered [17,35]. Omics data should deepen the question, not replace it [22].

Data integration also creates ethical and statistical challenges. High-dimensional metabolomics combined with wearables can generate false discoveries, privacy concerns, and misleading individualized recommendations if models are not validated. Athletes may be exposed to unnecessary anxiety or training restriction if complex data are presented as deterministic risk scores. Therefore, metabolomic integration should be transparent, interpretable, and evaluated against clinically or performance-relevant outcomes [22].

### 11.6. Methodological Pitfalls in Sports Metabolomics

Metabolomics is methodologically demanding, and its value in athlete monitoring depends heavily on preanalytical control, metadata quality, analytical reproducibility, and transparent reporting. The National Institutes of Health (NIH) Common Fund Metabolomics Program and its Metabolomics Workbench infrastructure have emphasized the importance of data sharing, metadata, metabolite standards, protocols, tutorials, and analysis tools for improving reproducibility and reuse in metabolomics research [140]. In exercise studies, these principles are especially important because exercise is itself a major biological and preanalytical perturbation.

Preanalytical variables such as fasting status, diet, sample timing, tube type, anticoagulant, hemolysis, processing delay, centrifugation conditions, storage temperature, freeze–thaw cycles, exercise timing, hydration, menstrual-cycle phase, hormonal contraceptive use, and circadian rhythm can strongly influence measured metabolites [141,142]. Kirwan et al. [141] emphasized that metabolomics samples should be collected, processed, transported, and stored in reproducible conditions, and that less obvious variables (i.e., vial material, batch, time to freezer, sample volume, centrifuge speed, storage duration, and environmental conditions during collection) can affect data validity. Dunn et al. [142] remains a foundational protocol for large-scale metabolic profiling of serum and plasma, but more recent preanalytical guidance reinforces the need to report and control the full sample pathway from participant preparation to biobanking [141,142].

In athlete studies, preanalytical control should start before the blood or urine sample is collected. The protocol should specify recent training, time since last exercise bout, fasting duration, pre-test meal, carbohydrate availability, fluid intake, caffeine, alcohol, supplements, medication, illness, sleep, travel, heat exposure, altitude exposure, menstrual-cycle phase, hormonal contraceptive use, and time of day [21,25,141]. Without this information, metabolomic differences may reflect uncontrolled preparation rather than meaningful differences in training adaptation, fatigue, or recovery.

Reporting standards are also essential. Sumner et al. [143] proposed minimum reporting standards for chemical analysis in metabolomics, including experimental design, analytical procedures, metabolite identification, and quality control. In sports metabolomics, essential metadata should include exercise protocol, sampling timing, diet, fasting duration, training status, sex, menstrual-cycle or contraceptive status, recent illness, sleep, hydration, supplements, medications, and environmental conditions [133,140,144].

Multiple testing and model overfitting are major risks. Broadhurst and Kell [144] emphasized statistical strategies for avoiding false discoveries in metabolomics, an issue that becomes especially important when sample sizes are small and metabolite features are numerous. Athlete studies often have limited sample sizes because elite cohorts are difficult to recruit, making external validation, appropriate cross-validation, transparent preprocessing, and prespecified analytical decisions essential [144].

Metabolite identification is another bottleneck. Unknown features may be statistically associated with exercise but biologically uninterpretable if they are not confidently annotated. Pang et al. [145] described MetaboAnalyst as a platform for metabolomics data analysis and interpretation, while Wishart et al. [146] described Human Metabolome Database (HMDB) 5.0 as a major knowledge resource for human metabolites. These tools are useful for pathway interpretation, but they do not replace biochemical validation or metabolite identification confidence [145,146].

Data sharing and repository-based curation can improve reproducibility. Salek et al. [147] highlighted the importance of metabolomics repositories, metadata, and standardization for transparency and reuse, while Metabolomics Workbench provides an NIH Common Fund-supported infrastructure for depositing metabolomics data and associated metadata [140,147]. Platform selection also shapes interpretation: NMR offers high reproducibility and relatively simple preparation but lower sensitivity for many low-abundance metabolites, whereas LC-MS provides broader coverage of lipids, acylcarnitines, bile acids, oxylipins, and low-abundance molecules but requires strong quality control [142,148].

For athlete monitoring, the most important methodological principle is restraint. Metabolomics can reveal rich biological patterns, but applied recommendations should not exceed the maturity of the evidence [22]. The strongest current use is mechanistic interpretation and hypothesis generation; routine training decisions based on metabolomic panels require more longitudinal validation, sport-specific reference data, standardized preanalytical procedures, and demonstration of incremental value beyond simpler tools [4,22].

## 12. Sport-Specific Interpretation

Having moved from domain-level biomarkers to systems-level metabolic interpretation, the next layer is sport context. Sport-specific interpretation is essential because the same biomarker may have different meanings depending on the dominant metabolic demands, mechanical load, environmental exposure, competition structure, body-composition pressures, and recovery constraints of the sport. For example, creatine kinase (CK) values that may appear alarming in a sedentary clinical context can fall within athlete-specific distributions, with Mougios [149] reporting reference intervals of 82–1083 U/L in male athletes and 47–513 U/L in female athletes. Such values should not be treated as universal safe or unsafe thresholds, but they illustrate why athlete-specific context matters [4,149].

A useful sport-specific approach does not classify sports rigidly, because many sports combine endurance, strength, speed, skill, contact, heat exposure, and weight regulation. Instead, biomarkers should be prioritized according to the dominant physiological question: intensity prescription, substrate use, muscle damage, hydration, iron status, endocrine stress, low energy availability, inflammation, illness risk, or return-to-play readiness [5,25]. In this sense, sport-specific interpretation is not a separate layer added after biomarker analysis; it is the condition that determines whether a biomarker is meaningful at all.

### 12.1. Endurance Sports

Endurance sports such as distance running, cycling, rowing, triathlon, cross-country skiing, and open-water swimming typically impose high cumulative metabolic load, large energy expenditure, repeated glycogen depletion, fluid and electrolyte challenges, and sustained cardiovascular and mitochondrial demand. In these athletes, lactate thresholds, critical power or speed, substrate oxidation, glucose availability, free fatty acids, ketone bodies, acylcarnitines, urea/BUN, ferritin, hemoglobin, hepcidin, cortisol, and hydration markers may all be relevant depending on the monitoring question [37,39,150].

Training intensity distribution influences which biomarkers are most informative. In one comparative study of well-trained endurance athletes, Stöggl and Sperlich [151] reported greater improvements in several key endurance outcomes with polarized training than with threshold, high-intensity, or high-volume approaches. However, this finding should not be generalized as evidence that polarized training is universally superior; the optimal distribution of low-, moderate-, and high-intensity work remains context-dependent and debated across athlete level, sport, training phase, outcome, and program design [152]. Accordingly, lactate, RER, HR, HRV, perceived exertion, and substrate-related metabolites should be interpreted against the intended training-intensity distribution rather than isolated sessions [151,152].

Tapering also changes biomarker interpretation. Mujika and Padilla [153] described tapering as a progressive reduction in training load intended to reduce accumulated fatigue while maintaining adaptation. During tapering, reductions in CK, urea, cortisol, perceived fatigue, or inflammatory markers may reflect restoration of homeostasis rather than detraining if performance readiness improves [31,153]. Conversely, persistent biomarker disturbance despite tapering may suggest unresolved fatigue, illness, inadequate fueling, or excessive prior load.

Endurance sports are also where metabolic flexibility markers are most often used, but they require nuance. Combined measurement of blood lactate, fat oxidation, and carbohydrate oxidation during graded exercise has been proposed as an indirect way to assess metabolic flexibility and mitochondrial function [150]. However, high fat oxidation or low lactate at a given workload is not always favorable: it may reflect improved oxidative capacity, but also low carbohydrate availability, glycogen depletion, or inability to sustain high glycolytic flux when required [45,150]. Similarly, β-hydroxybutyrate may rise with prolonged exercise, fasting, low carbohydrate availability, or exogenous ketone ingestion, but ketone availability does not automatically translate into improved performance [78].

High-intensity endurance training adds another layer. Laursen [154] reviewed the role of high-intensity training in highly trained endurance athletes, highlighting that intense sessions may be necessary to improve performance but also increase glycolytic flux, sympathetic activation, lactate production, purine degradation, and neuromuscular strain. Thus, a high-intensity interval block may transiently increase lactate, CK, hypoxanthine, ammonia, cortisol, and perceived fatigue without indicating maladaptation if recovery and performance trends remain appropriate [48,154].

### 12.2. Strength and Power Sports

Strength and power sports such as weightlifting, powerlifting, sprinting, jumping, throwing, bodybuilding, and many explosive field events are characterized by high force production, phosphocreatine turnover, neuromuscular stress, mechanical tension, eccentric loading, and sometimes substantial muscle damage. In these contexts, CK, LDH, myoglobin, creatinine, testosterone, cortisol, uric acid, ammonia, acylcarnitines, and selected amino acid markers may be especially relevant [9,52].

Suchomel et al. [155] emphasized the importance of muscular strength for athletic performance across jumping, sprinting, change of direction, and sport-specific tasks. For biomarker interpretation, this means that muscle damage markers must be related to the intended neuromuscular stimulus. A CK rise after heavy eccentric squatting or plyometrics may be expected, whereas the same CK rise after a low-load technical session may require a different interpretation [9,155].

Schoenfeld [156] described mechanical tension, metabolic stress, and muscle damage as major mechanisms implicated in hypertrophy-oriented resistance training. These mechanisms are metabolically relevant because they can modify lactate, H^+^, Pi, ROS/RNS, amino acid turnover, mechanistic target of rapamycin (mTOR)-related signaling, inflammatory lipid mediators, and repair pathways [156]. Therefore, strength-sport biomarkers should not focus only on CK; they should also consider whether metabolic stress, protein synthesis, recovery, and performance quality are aligned with the training goal [156].

Concurrent training complicates interpretation because endurance and strength stimuli may produce partly divergent molecular adaptations. Nader [157] reviewed molecular aspects of concurrent strength and endurance training, emphasizing that adaptation specificity involves distinct signaling pathways. In athletes combining endurance and resistance training, CK, lactate, acylcarnitines, amino acids, cortisol, testosterone, and substrate markers may reflect interference, sequencing, inadequate recovery, or simply the combined stress of two valid stimuli [157].

### 12.3. Team, Intermittent, and Contact Sports

Team and intermittent sports such as football, basketball, handball, rugby, hockey, futsal, and racquet sports combine repeated accelerations, decelerations, changes of direction, collisions or contacts, high-speed running, technical actions, and incomplete recovery. Bangsbo et al. [158] described elite football as a sport with substantial intermittent metabolic demand, where players perform repeated high-intensity actions superimposed on prolonged activity. Stølen et al. [159] similarly emphasized the mixed aerobic–anaerobic nature of soccer physiology. This means that lactate, CK, neuromuscular tests, GPS-derived load, HR/HRV, RPE, hydration, and inflammatory markers must be interpreted within match demands [158,159].

Post-match fatigue is multidimensional. Nédélec et al. [160] reviewed recovery after soccer match play, describing fatigue mechanisms involving glycogen depletion, muscle damage, inflammation, dehydration, sleep disturbance, and neuromuscular impairment. Therefore, a post-match biomarker panel showing elevated CK, CRP, urea, cortisol, altered lactate kinetics, or reduced neuromuscular performance may reflect expected match-induced fatigue rather than pathology, particularly during congested fixtures [160].

Repeated-sprint ability adds further complexity. Bishop et al. [161] emphasized that repeated-sprint performance depends on phosphocreatine resynthesis, glycolytic contribution, oxidative recovery, ion regulation, H^+^, Pi, K^+^, neuromuscular factors, and recovery kinetics. Buchheit and Laursen [162,163] also highlighted that high-intensity interval training can be programmed to emphasize cardiopulmonary, anaerobic, or neuromuscular load. Thus, lactate or CK alone cannot capture the biological load of intermittent sports; the pattern across sprint decrement, neuromuscular performance, HR response, RPE, and metabolic markers is more informative [161,162,163].

High-intensity intermittent exercise is relevant for metabolomics because it can generate large but transient perturbations in lactate, pyruvate, succinate, purine metabolites, ammonia, catecholamine-sensitive lipolysis, and N-lactoyl-amino acids [35,48]. One example is N-lactoyl-phenylalanine (Lac-Phe), an exercise-inducible metabolite formed from lactate and phenylalanine that increases after vigorous exercise and has been implicated in appetite regulation and energy balance, mainly in preclinical work and early human studies [137,164]. However, Lac-Phe should currently be considered an exploratory exercise-responsive metabolite rather than an applied athlete-monitoring biomarker. There are no established athlete reference values, sport-specific response ranges, or validated individual decision thresholds, and its meaning may depend on exercise intensity, feeding status, phenotype, analytical platform, and sampling timing [137,164]. Its main value in this review is therefore conceptual: it illustrates that intense exercise can generate signaling metabolites beyond traditional markers, not that Lac-Phe should guide training, recovery, or body-composition decisions in athletes.

Contact and collision sports require additional caution because trauma-related inflammation and muscle damage are common. A rugby, combat-sport, or collision-sport athlete may show high CK and CRP after competition due to impacts, eccentric actions, or tissue trauma rather than systemic illness [10,52]. However, interpretation should change when elevations are accompanied by focal pain, swelling, concussion symptoms, fever, dark urine, reduced urine output, or persistent performance decline [63,64].

### 12.4. Aesthetic, Weight-Sensitive, Combat, and Indoor Sports

Weight-sensitive and aesthetic sports create specific biomarker risks because performance, selection, or judging may reward low body mass, low fat mass, or a particular physique. Combat sports add acute weight-making practices, dehydration, glycogen manipulation, sodium restriction, and rapid post-weigh-in refueling [165,166]. Franchini et al. [165] reviewed rapid weight loss in combat sports and described potential physiological, psychological, and performance consequences. Reale et al. [166] emphasized that acute weight loss and recovery nutrition should be individualized and planned rather than improvised close to competition.

In these sports, biomarkers such as urea/BUN, creatinine, Na^+^, K^+^, urine specific gravity, osmolality, glucose, β-hydroxybutyrate, cortisol, testosterone, thyroid markers, leptin, ferritin, and bone markers may reflect weight manipulation, dehydration, low energy availability, or recovery failure rather than training adaptation [17,70]. A rise in BUN and creatinine before weigh-in, for example, may reflect dehydration and reduced renal perfusion rather than high training stress [70,72]. Similarly, low glucose or high β-hydroxybutyrate may reflect carbohydrate restriction, fasting, or rapid weight loss rather than superior metabolic flexibility [45,78].

Sundgot-Borgen and Garthe [167] highlighted the challenge of body weight and body composition in aesthetic and Olympic weight-class sports. In such contexts, biomarker interpretation should actively screen for RED-related patterns, including low energy availability, menstrual dysfunction, low testosterone, low T3, low leptin, impaired bone turnover, recurrent injury, low ferritin, or poor recovery [17,167]. The goal is not merely to monitor performance but to prevent medical normalization of under-fueling [17,167].

Female athletes require specific attention to reproductive physiology. McNulty et al. [168] reported that exercise performance may be trivially reduced in the early follicular phase compared with other menstrual-cycle phases, but evidence quality was low and individual responses varied. For biomarker interpretation, this means that menstrual-cycle phase, hormonal contraception, amenorrhea, oligomenorrhea, and reproductive status should be recorded when interpreting endocrine, fluid, thermoregulatory, substrate, and performance markers [125,168].

Indoor sports may increase risk of vitamin D inadequacy because sunlight exposure is limited. Owens et al. [169] reviewed vitamin D in athletes, noting potential relevance to muscle, bone, and immune function. Close et al. [170] observed inadequate vitamin D concentrations in United Kingdom-based athletes during winter months, supporting seasonal monitoring in at-risk athletes. In indoor, aesthetic, and weight-sensitive sports, vitamin D should be interpreted with season, sunlight exposure, bone stress injury history, calcium intake, menstrual status, energy availability, and training load; in these contexts, clinical deficiency thresholds may need to be complemented by athlete-specific, bone-health-oriented interpretation [95,169,170].

Sports nutrition guidelines emphasize that athletes require individualized strategies for energy, carbohydrate, protein, fluid, and micronutrient intake. Burke et al. [171] highlighted the importance of carbohydrate availability for training and competition, while Thomas et al. [172] provided broader nutrition recommendations for athletic performance. In sport-specific biomarker interpretation, these nutrition principles determine whether changes in lactate, glucose, urea, ketones, cortisol, iron markers, or recovery indicators represent adaptation, acute manipulation, or inadequate fueling [171,172].

### 12.5. Matching Biomarkers to Sport-Specific Questions

Across sports, the most useful biomarker is not necessarily the most sophisticated one, but the one that best matches the decision being made [4,25]. In endurance sports, lactate thresholds, substrate oxidation, ferritin, hepcidin, hydration markers, and carbohydrate-availability indicators may be central because intensity prescription, substrate availability, iron status, and fluid balance are common monitoring questions [37,39,88,150,151,152,153,154]. In strength and power sports, CK, LDH, myoglobin, creatinine, neuromuscular performance, and endocrine context may be more informative when the main concern is mechanical load, muscle damage, phosphocreatine turnover, or recovery from high-force training [9,52,61,62,63,155,156,157]. In team and intermittent sports, external load, repeated-sprint demands, CK, CRP, hydration, sleep, and neuromuscular readiness often need to be interpreted together because fatigue reflects the combined effects of sprinting, deceleration, collisions, glycogen depletion, inflammation, and congested fixtures [158,159,160,161,162,163]. In weight-sensitive and aesthetic sports, biomarkers should be especially sensitive to low energy availability, dehydration, endocrine suppression, iron deficiency, and bone-health risk [17,70,75,95,97,165,166,167,168,169,170,171,172].

Metabolomics adds value when it clarifies the biological nature of the sport stimulus. Lactate, pyruvate, succinate, purines, ammonia, and exploratory N-lactoyl-amino acid signals may be relevant after high-intensity exercise; glucose, NEFA, glycerol, β-hydroxybutyrate, and acylcarnitines may help characterize substrate use; and amino acids, oxylipins, kynurenine metabolites, bile acids, and microbiome-derived metabolites may provide insight into recovery, inflammation, and interorgan signaling [35,48]. However, these markers should be interpreted as pathway signals, not as validated diagnostic thresholds, readiness markers, or individual performance predictors.

A practical sport-specific approach is therefore to begin with the sport demand and the decision question, then choose the biomarker panel [4,25]. If the question is intensity prescription, lactate, ventilatory thresholds, critical power, and workload-specific responses are most relevant [37,38,39,40,150]. If the question is recovery from mechanical load, CK, myoglobin, soreness, jump performance, and muscle function are central [9,52,61,62,63]. If the question is substrate availability, glucose, lactate, RER, NEFA, glycerol, ketone bodies, and acylcarnitines may be useful [45,48,78,150]. If the question is health risk in weight-sensitive sports, endocrine, hematological, bone, hydration, and energy-availability markers should take priority [17,70,75,95,97,165,166,167,168,169,170,171,172].

**Figure 6:**
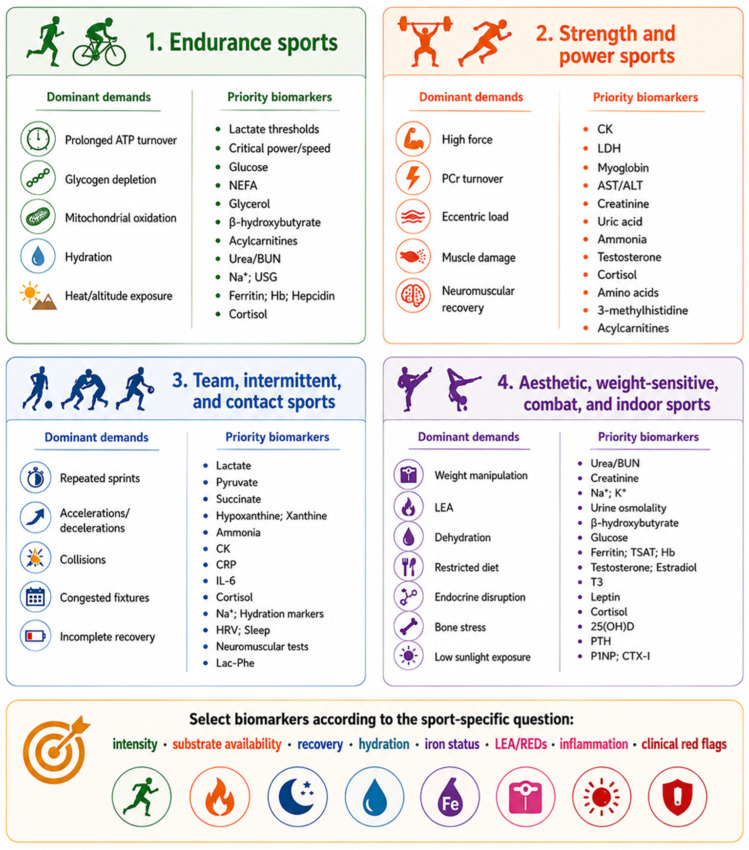
Sport-specific biomarker priorities according to dominant physiological demands. Biomarker selection should be guided by the dominant demands and decision questions of each sport context. Endurance sports often prioritize intensity prescription, substrate availability, hydration, iron status, and energy availability; strength and power sports emphasize muscle damage, phosphocreatine turnover, neuromuscular recovery, and anabolic–catabolic context; team, intermittent, and contact sports require integration of repeated-sprint metabolism, mechanical load, inflammation, hydration, sleep, and match congestion; and aesthetic, weight-sensitive, combat, and indoor sports require particular attention to low energy availability, acute weight manipulation, dehydration, endocrine suppression, iron status, vitamin D, and bone-health risk. These categories are heuristic rather than mutually exclusive, and biomarker panels should be adapted to the athlete, sport, training phase, and clinical or performance question.

A practical overview of sport-specific biomarker priorities according to dominant physiological demands and decision questions is provided in Figure 6.

## 13. Practical Framework for Decision-Making

The final interpretive layer is translation from biological information to proportional action. The practical value of athlete biomarkers depends on whether they improve decisions. A laboratory result should not automatically trigger action; it should refine a predefined question about training adaptation, recovery, nutritional status, hydration, illness, injury risk, or clinical safety [4,25]. Therefore, this section proposes a decision framework that integrates biomarker values, athlete-specific baselines, standardized sampling, sport demands, symptoms, performance, and selected interpretive anchors.

The values in Table 5 should be read as literature-informed orientation ranges, not clinical reference limits, athlete-specific reference intervals, universal cut-offs, or standalone decision thresholds. They are included to improve practical utility by showing values commonly reported in clinical, laboratory, and sport-science literature, but the article’s main argument remains that athlete interpretation should be contextual and longitudinal. Importantly, most values in the table are not derived from athlete-specific normative datasets. Some are based on general adult laboratory intervals, clinical conventions, or disease-oriented thresholds, whereas only selected entries, such as CK, include athlete-specific reference data [4,7,149]. Values may differ by laboratory method, sex, age, pubertal maturation, ethnicity, training status, muscle mass, sport, hydration, altitude, diet, illness, menstrual status, recent exercise, and assay platform [4,7]. Therefore, a value can be clinically “normal” yet abnormal for an athlete’s baseline, or outside a population interval yet expected in a given sport context [4,149].

### 13.1. Define the Question Before Ordering the Biomarker

The first decision rule is to define the question before ordering the test. A biomarker panel should be designed differently depending on whether the practitioner is asking about intensity prescription, muscle damage, hydration, iron status, illness, REDs risk, endocrine stress, or return-to-play readiness [4,25]. Without a prior question, broad testing increases the probability of incidental findings, overinterpretation, and unnecessary intervention [4].

For example, if the question is intensity prescription, lactate, ventilatory thresholds, critical power, and workload-specific responses may be appropriate [37,39]. If the question is neuromuscular recovery, CK, soreness, countermovement jump, sprint performance, and movement quality may be more relevant [9,175]. McGuigan et al. [175] emphasized that strength and power tests should be selected according to reliability, validity, sport relevance, and usefulness for program design. The same principle applies to biomarkers: select what can answer the question [175].

### 13.2. The BASE Model

Building on the four conceptual principles introduced in Section 3, BASE provides an applied mnemonic rather than a one-to-one relabeling of those principles: **B**aseline, **A**nalytical standardization, **S**port-specific context, and **E**vidence of functional change. The relationship between the two frameworks is deliberate. Baseline operationalizes athlete-specific comparison and includes the athlete’s prior biological profile; Analytical standardization establishes whether repeated measurements are sufficiently comparable; Sport-specific context situates the current result within both the demands of the sport and the athlete’s longitudinal exposure history, including training phase, competition schedule, prior responses to comparable loads, recent transitions, injury or illness, travel, altitude, heat, and recovery context; and Evidence of functional change is the triangulation step in which the preceding information is integrated with symptoms, recovery, internal load, and objective performance [4,25,31].

Longitudinal trend is therefore not removed from the applied model or replaced by Sport-specific context. It is a temporal dimension that informs Baseline and Analytical standardization and becomes especially explicit within Sport-specific context, where the present biomarker value is interpreted against the athlete’s trajectory and prior response to comparable exposures. Likewise, Evidence of functional change does not refer to a single performance test or symptom in isolation; it represents convergence or meaningful discordance between the biomarker signal and the evidence assembled under B, A, and S. In this structure, Section 3 provides the conceptual filters, BASE organizes their applied interpretation, and the green–yellow–red logic translates the resulting degree of convergence, severity, and trajectory into proportional action. BASE is intended to reduce interpretive error, not to generate automatic decisions.

Evidence of functional change can come from simple but reliable measures, but no single measure constitutes triangulation by itself. Claudino et al. [176] found that countermovement jump variables can be useful for monitoring neuromuscular status, although protocols and interpretation must be standardized. Heart-rate variability (HRV) may also provide information on autonomic regulation, but Plews et al. [177] and Bellenger et al. [178] emphasized that HRV interpretation requires individualized trends and context rather than isolated values. These tools complement biomarkers because their value emerges from convergence with the athlete’s baseline, longitudinal trajectory, symptoms, recovery, and sport-specific demands [176,177,178].

The BASE model also reduces false alarms. A high CK value without performance loss, soreness, or symptoms may be a yellow signal rather than a red signal. Conversely, a normal CK value with poor sleep, high rating of perceived exertion (RPE), reduced jump performance, and declining pace may still indicate impaired recovery [4,16]. The model therefore protects against both biochemical overreaction and functional under-recognition [4,176].

### 13.3. Green–Yellow–Red Decision Logic

After interpretation through BASE, findings can be translated into a proportional green–yellow–red response according to convergence, severity, and trajectory. A green signal indicates that biomarkers are consistent with baseline or expected training response, sampling was standardized, performance is stable, and symptoms are absent. In this case, the plan can usually be maintained [4,25]. A yellow signal indicates uncertainty: a marker is altered, but sampling was suboptimal, symptoms are absent, or performance is stable. The appropriate response is usually to repeat, monitor, and review contextual factors [4,25].

A red signal indicates convergence of abnormal biomarker trends with clinical symptoms, poor recovery, or objective performance decline. Gabbett [179] emphasized that injury risk relates not simply to high training load but to inappropriate load relative to athlete capacity. Impellizzeri et al. [180] clarified that external load and internal load are distinct constructs, with internal load reflecting the psychophysiological response to external work. Therefore, red signals should be based on convergence between biological disturbance, internal load, external load, and functional impairment [179,180].

**Figure 7:**
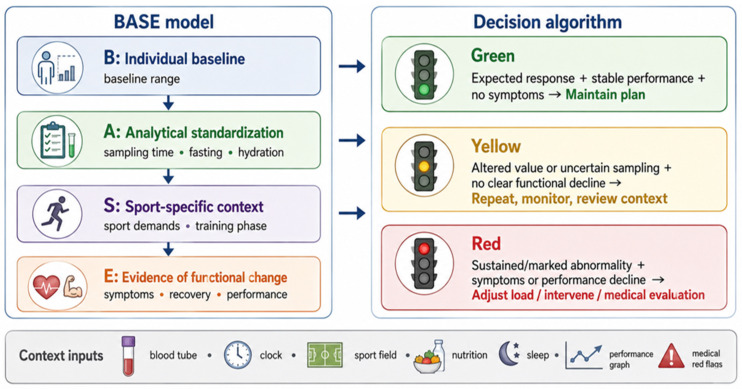
BASE model and green–yellow–red decision algorithm for athlete biomarker interpretation. The BASE model structures biomarker interpretation through four sequential domains: individual baseline, analytical standardization, sport-specific context, and evidence of functional change. Longitudinal trend is treated as a temporal dimension across the model, particularly through comparison with the athlete’s prior profile and sport-specific exposure history, whereas evidence of functional change represents triangulation of the preceding information with symptoms, recovery, and performance. These domains inform a proportional decision algorithm in which green signals support maintaining the current plan, yellow signals suggest repeating or confirming the finding and monitoring the context, and red signals indicate the need for training adjustment, intervention, or medical evaluation. Contextual inputs such as sampling conditions, sport demands, nutrition, sleep, performance data, and clinical red flags should be considered before translating biomarker results into action.

This decision logic is not meant to replace clinical judgment. It is a proportionality model: the level of action should match the convergence, severity, and trajectory of signals [4,25]. A mild isolated CRP elevation after a match may require observation; CRP elevation with fever and malaise may require medical evaluation [102]. A high CK after eccentric training may be expected; high CK with severe pain, dark urine, reduced urine output, or rising creatinine is different [63,64]. The BASE logic and green–yellow–red decision process are summarized in Figure 7.

### 13.4. Red Flags Requiring Medical Evaluation

Clinical recommendations in this review are intentionally limited to escalation principles rather than treatment protocols or diagnostic thresholds. Certain patterns should move the decision from athlete monitoring to medical evaluation: an isolated abnormal result without compatible symptoms, particularly when sampling conditions are uncertain, should generally prompt contextual reassessment and, when appropriate, repeat testing under standardized conditions. Persistent or worsening abnormalities that converge with unexplained performance decline, recurrent symptoms, weight loss, menstrual dysfunction, impaired recovery, or other functional changes warrant clinician-led evaluation. Acute systemic, renal, neurological, cardiopulmonary, or severe muscle symptoms should prompt timely medical assessment irrespective of whether a single biomarker crosses a predefined threshold [17,63,64,70]. These principles are not diagnostic criteria and do not replace clinical judgment, laboratory-specific interpretation, or appropriate referral pathways.

Foster [181] emphasized that monitoring training responses can help identify excessive strain and illness risk, while Hooper and Mackinnon [182] highlighted the importance of monitoring fatigue, mood, sleep, and stress in relation to overtraining. Kenttä and Hassmén [183] conceptualized overtraining and recovery as a balance between stress and restoration, supporting the idea that biomarker decisions should consider total stress rather than training alone. These perspectives remain relevant because many red flags emerge from cumulative stress, not a single abnormal result [181,182,183].

Budgett [184] described underperformance and fatigue in athletes as complex syndromes requiring broad differential diagnosis. This is particularly relevant when an athlete presents with persistent fatigue, normal routine biomarkers, and declining performance: nutritional, infectious, endocrine, psychological, sleep-related, and medical causes should all be considered [15,184]. Similarly, Hainline et al. [185] emphasized that mental health symptoms in elite athletes require recognition and appropriate support, not reduction to physiological markers alone [185].

A conservative rule is appropriate: when biomarker abnormalities are accompanied by systemic symptoms, neurological symptoms, renal signs, severe pain, unexplained weight loss, disordered eating risk, menstrual dysfunction, chest pain, exertional collapse, or persistent underperformance, the case should be escalated beyond routine sport-science monitoring [17,63,64,70,185]. Biomarkers can support recognition, but they should not delay clinical evaluation when red flags are present [63,64,185].

### 13.5. Common Misinterpretations and How to Avoid Them

A common misinterpretation is treating reference intervals as performance thresholds. A biomarker can be inside the population reference range and still be abnormal for an athlete if it deviates substantially from baseline. Conversely, a value outside the population range may be expected after a particular sport exposure [4,149]. This applies especially to CK, creatinine, ferritin, cortisol, and inflammatory markers [4,149].

A second error is interpreting biomarkers without time course. CK, myoglobin, CRP, cortisol, hepcidin, lactate, glucose, and many metabolites have different kinetics [10,48]. A sample taken immediately post-exercise, 24 h later, or after a rest day may reflect different underlying biology. Therefore, longitudinal interpretation requires repeated sampling under comparable conditions [25,26].

A third error is assuming that more biomarkers always improve decision-making. Large panels increase complexity and the likelihood of incidental abnormalities. The appropriate question is whether the biomarker adds information beyond symptoms, training load, performance tests, diet, sleep, and clinical history [4,22]. In some cases, a simple panel measured consistently may be superior to a broad omics panel measured inconsistently [22,132].

A fourth error is confusing association with actionability. Even if a metabolite or hormone is associated with fatigue, adaptation, or performance, it may not yet be validated for individual decisions [20,22]. Actionability requires that the marker improves interpretation, changes a decision, or identifies a risk that would not otherwise be recognized using simpler tools [4,22]. This is especially important for emerging metabolomic markers, where statistical associations may precede clinical or sport-specific validation [20,21,22].

Finally, biomarkers should not be used to bypass the athlete narrative. Symptoms, training history, nutrition, sleep, psychological stress, menstrual or reproductive context, travel, illness exposure, and performance trajectory remain essential. A biomarker result should sharpen interpretation, not replace reasoning. The safest practical approach is to ask whether the laboratory signal is biologically plausible, methodologically valid, consistent over time, and functionally meaningful [4,25].

## 14. Limitations of the Evidence and Future Research

The evidence on athlete biomarkers is heterogeneous because studies differ substantially in sport modality, training status, sex distribution, competitive level, sampling timing, nutritional control, analytical platform, and outcome definition. Many biomarkers discussed in this review are biologically plausible and mechanistically informative, but their translation into individual athlete decisions remains uneven. Routine markers such as CK, ferritin, CRP, cortisol, or lactate are widely used but nonspecific, whereas emerging metabolomic markers such as acylcarnitines, kynurenine metabolites, bile acids, oxylipins, BAIBA, or Lac-Phe are promising but not yet sufficiently validated for routine monitoring. A major limitation of the field is that many studies are cross-sectional, acute-exercise based, or conducted in small and highly selected samples, making it difficult to establish robust athlete-specific reference intervals, causal pathways, or decision thresholds.

This review also has limitations. As a narrative review, it aimed to integrate physiological, biochemical, metabolomic, and applied sport-science evidence rather than to answer a narrowly defined question using systematic review methods. Therefore, study selection was interpretive rather than exhaustive, and no formal risk-of-bias assessment, meta-analysis, or certainty-of-evidence grading was performed. The breadth of the topic also required prioritizing conceptual integration over detailed coverage of every biomarker, sport, assay, or clinical scenario. Consequently, the proposed frameworks, tables, and decision models should be interpreted as practical synthesis tools rather than validated algorithms. In particular, the BASE model and the green–yellow–red decision logic have not been prospectively validated to determine whether their use improves athlete outcomes, reduces false alarms, prevents missed clinical problems, or adds incremental value beyond existing monitoring practices. They are intended to support structured reasoning, not to replace clinical evaluation, individualized coaching, nutrition assessment, laboratory-specific interpretation, or shared decision-making with qualified professionals.

Future research should move from isolated biomarker associations toward longitudinal, within-athlete designs that test whether biomarker-informed decisions improve recovery, adaptation, health, and performance beyond simpler monitoring tools. Studies should standardize preanalytical conditions, report nutrition, hydration, sleep, menstrual-cycle or contraceptive status, recent training load, illness, medication, supplements, and environmental exposure, and include repeated measures across meaningful training periods. There is a particular need for sex-specific, sport-specific, and age-specific models; external validation of metabolomic and multi-omic panels; integration with wearables and performance tests; and prospective evaluation of decision frameworks such as BASE and green–yellow–red logic. The next step for the field is not merely to discover more biomarkers, but to determine when a biomarker or framework changes a decision, improves an outcome, reduces harm, or provides incremental value beyond existing monitoring systems.

## 15. Conclusions

Biomarkers in athletes are dynamic biological signals whose meaning depends on individual baseline, sampling conditions, temporal trajectory, sport demands, and functional state. Routine markers such as lactate, creatine kinase, urea/BUN, creatinine, electrolytes, ferritin, C-reactive protein, cortisol, testosterone, and vitamin D can provide useful information, but no single value should be assumed to represent adaptation, maladaptation, or clinical risk without considering these dimensions.

A metabolically informed approach improves interpretation by linking routine biomarkers to broader physiological pathways. Lactate should be understood not only as an intensity marker, but also as a fuel, shuttle metabolite, and signaling molecule. Urea and creatinine should be interpreted through nitrogen metabolism, protein intake, hydration, renal perfusion, and muscle mass. Ferritin and transferrin saturation require integration with inflammation and hepcidin biology. CRP, cytokines, kynurenine metabolites, short-chain fatty acids, bile acids, and indoles reflect immunometabolic crosstalk rather than single disease states. Emerging metabolomics may help characterize pathway-level patterns of substrate stress, muscle damage, inflammatory signaling, low energy availability, and recovery, but it should complement, not replace, routine markers and functional monitoring.

For practice, biomarker interpretation should follow a structured and proportional decision logic. The biological question should be defined before ordering a test; sampling should be standardized; values should be compared with individual baselines; longitudinal trends should be prioritized over isolated measurements; and decisions should be anchored to performance, symptoms, recovery, nutrition, and training load. The proposed BASE model and green–yellow–red decision logic may support this reasoning by helping practitioners distinguish expected responses, uncertain findings requiring reassessment, and red flags requiring intervention or medical evaluation.

Overall, biomarker values gain interpretive power when they are anchored to the athlete’s history, sport context, symptoms, nutritional and recovery status, and functional performance. Future monitoring studies should therefore move beyond isolated associations and test whether biomarker-informed frameworks improve decisions, reduce harm, and add value beyond simpler tools already available in sport science, sports medicine, and nutrition practice.

## Figures and Tables

**Table 1:** Core principles for contextual biomarker interpretation in athletes.

Principle	Main Question	Practical Implication	Common Error Avoided
Individual baseline	Is this value unusual for this athlete?	Compare with athlete-specific values collected during stable periods	Overreliance on population reference ranges
Sampling standardization	Was this sample collected under comparable conditions?	Control or record time of day, exercise timing, nutrition, hydration, sleep, illness, supplements, and menstrual-cycle context	Comparing biologically different states
Longitudinal trend	Is the change sustained and larger than expected variation?	Evaluate direction, magnitude, kinetics, repeated measurements, and prior responses to comparable sport exposures	Overreacting to isolated values
Functional triangulation	Does the biomarker converge with performance, symptoms, recovery, and load data?	Integrate the biomarker signal with baseline, sampling validity, sport-specific trajectory, symptoms, recovery, and performance	Treating nonspecific biomarkers as diagnostic tests

**Table 2:** Metabolite-centered interpretation of exercise intensity and metabolic flexibility in athletes.

Biomarker or Metabolite Class	Main Biological Interpretation	Typical Exercise-Related Pattern	Key Contextual Modifiers	Main Interpretive Risk
Lactate	Glycolytic flux, pyruvate handling, lactate production–clearance balance	Rises with increasing intensity; may stabilize below maximal lactate steady state (MLSS)/critical thresholds	Training status, glycogen availability, sampling timing, modality, active recovery	Interpreting lactate as “anaerobic waste” or as fatigue itself
Pyruvate	End product of glycolysis and substrate for mitochondrial oxidation or lactate production	Increases with glycolytic flux and carbohydrate use	Oxygen delivery, mitochondrial capacity, LDH activity, NAD^+^/NADH state	Treating plasma pyruvate as a direct proxy for intramuscular flux
Glucose	Circulating carbohydrate availability	Maintained, rises, or falls depending on intensity, duration, hepatic output, and intake	Feeding, glycogen, catecholamines, insulin, carbohydrate ingestion	Assuming normal glucose excludes low muscle glycogen
Non-esterified fatty acids (NEFA)/free fatty acids	Lipolysis and circulating lipid substrate availability	Higher at lower intensities and prolonged exercise; may fall during high intensity	Insulin, catecholamines, carbohydrate intake, fasting, sex, training status	Equating high NEFA availability with high oxidation
Glycerol	Lipolysis marker	Usually rises with prolonged exercise and catecholamine stimulation	Fasting, adipose tissue lipolysis, exercise duration, temperature	Interpreting as direct fat oxidation rather than lipolysis
β-hydroxybutyrate/acetoacetate	Hepatic ketogenesis and alternative oxidative fuel availability	May rise with prolonged exercise, fasting, low carbohydrate availability, or supplementation	Diet, fasting, carbohydrate intake, exercise duration, recovery	Equating ketosis with improved performance
Acylcarnitines	Mitochondrial fatty acid transport, β-oxidation flux, incomplete oxidation	Species-specific changes with intensity, duration, and recovery	Diet, fasting, carnitine availability, mitochondrial function, training status	Calling them simple “fat-burning” markers
TCA intermediates	Central carbon flux and mitochondrial substrate handling	Citrate, succinate, malate, fumarate may change with exercise intensity and recovery	Sampling matrix, timing, oxygen availability, mitochondrial flux	Overinterpreting plasma levels as direct TCA cycle rate
Purine metabolites	ATP turnover and adenine nucleotide degradation	Hypoxanthine, xanthine, uric acid, inosine may rise after high-intensity or prolonged exercise	Intensity, hypoxia, recovery time, renal clearance	Interpreting them only as oxidative stress markers
Succinate	TCA intermediate and potential signaling metabolite	May rise with intense exercise and metabolic stress	Intensity, hypoxia, mitochondrial redox state, sampling timing	Treating it as a validated routine monitoring marker
Lac-Phe	N-lactoyl-amino acid linked to high lactate flux and appetite/metabolic signaling	Increases especially after vigorous or sprint-type exercise	Intensity, lactate production, phenotype, feeding, analytical platform	Premature use as a performance or weight-loss biomarker
RER-derived carbohydrate/fat oxidation	Whole-body substrate oxidation estimate	Fat oxidation predominates at lower intensities; carbohydrate oxidation rises with intensity	Steady-state validity, hyperventilation, diet, stage duration	Applying equations during non-steady-state or high-intensity exercise

**Table 3:** Explicit interpretation of hematological and iron-related biomarkers in athletes.

Marker	Main Biological Meaning	Pattern Suggesting Concern	Key Confounders	Interpretation Note
Hb	Oxygen-carrying capacity per blood volume	Low Hb with symptoms, low ferritin/TSAT, high sTfR	Plasma volume, hydration, altitude, sex	Concentration, not total Hb mass
Hct	Erythrocyte fraction of blood volume	Low Hct with iron-restricted profile or anemia symptoms	Plasma volume expansion, dehydration, heat acclimation	Low Hct may reflect pseudoanemia
MCV/MCH	Red cell size and hemoglobin content	Low values suggest microcytosis/hypochromia	Recent iron therapy, mixed deficiencies	Late markers compared with ferritin/TSAT
Ferritin	Iron stores; acute-phase reactant	Low ferritin suggests depleted stores	Inflammation, infection, liver stress, muscle damage	Normal ferritin does not exclude functional restriction
Serum iron	Circulating iron concentration	Low with low TSAT may suggest restricted availability	Diurnal variation, diet, inflammation, recent exercise	Weak alone; interpret with transferrin/TSAT
Transferrin/total iron-binding capacity (TIBC)	Iron transport capacity	High with iron deficiency; low with inflammation	Inflammation, nutrition, liver function	Needed for TSAT calculation
TSAT	Transferrin-bound iron availability	Low TSAT suggests low circulating iron availability	Inflammation, diurnal variation, recent diet	Useful when ferritin is ambiguous
sTfR	Cellular iron demand/iron-deficient erythropoiesis	High sTfR suggests tissue iron demand	Erythropoiesis, hemolysis, assay variability	Less affected by inflammation than ferritin
Hepcidin-25	Master regulator of iron export and absorption	High post-exercise hepcidin may reduce absorption	IL-6, iron stores, time of day, exercise timing	Promising but not routine in most settings
CRP	Inflammatory context for iron markers	Elevated CRP complicates ferritin interpretation	Infection, muscle damage, illness	Helps detect pseudo-normal ferritin
Reticulocyte Hb	Recent iron availability for erythropoiesis	Low values suggest iron-restricted erythropoiesis	Assay availability, recent therapy	Useful early functional marker

**Table 4:** Endocrine biomarkers in athlete monitoring: interpretation and major confounders.

Biomarker	Main Endocrine Axis	Possible Interpretation in Athletes	Key Confounders	Practical Caution
Cortisol	Hypothalamic–pituitary–adrenal (HPA) axis	Total stress load, prolonged exercise, low carbohydrate availability, sleep disruption	Circadian rhythm, sleep, travel, illness, psychological stress, sample matrix	Not diagnostic of overtraining
Testosterone	Hypothalamic–pituitary–gonadal (HPG) axis	Anabolic-reproductive status, chronic endurance adaptation, recovery context	SHBG, sleep, illness, energy availability, assay quality	Low value requires symptoms and repeat testing
Testosterone-to-cortisol (T:C) ratio	HPA–HPG interaction	Possible strain or altered anabolic-catabolic context	Changes in either hormone, timing, assay variability	Do not use as a standalone traffic light
Estradiol/progesterone	Ovarian axis	Menstrual function, bone and metabolic context	Cycle phase, contraceptives, reproductive status	Requires reproductive-history context
LH/FSH	Hypothalamic-pituitary-gonadal regulation	Reproductive-axis suppression in LEA/REDs	Pulsatility, cycle phase, sampling frequency	Single values may be misleading
T3/T4/TSH	Thyroid axis	Metabolic adaptation, energy conservation, thyroid disease context	Energy availability, illness, assay timing	Low T3 may reflect adaptation to LEA
Leptin	Adipose-energy signaling	Energy stores, acute/chronic energy deficiency	Fat mass, sex, energy intake, circadian rhythm	Not a routine performance marker
Insulin	Pancreatic-substrate regulation	Carbohydrate availability, energy status, metabolic health	Feeding, timing, exercise, insulin sensitivity	Interpret with glucose and diet
IGF-1	GH–IGF axis	Anabolic and bone-metabolic context	Energy availability, age, sleep, training, illness	Low values may reflect LEA or systemic illness

**Table 5:** Selected literature-informed interpretive anchors for common athlete biomarkers. Values are orientation ranges to support contextual interpretation, not universal clinical decision thresholds or athlete-specific reference intervals unless explicitly stated. Source context indicates whether the interpretive anchor is primarily derived from general adult/clinical data, exercise-specific, or mixed evidence. “Mixed evidence” indicates that a general clinical or laboratory anchor is interpreted alongside athlete- or exercise-specific evidence rather than derived from a validated athlete-specific reference interval.

Marker	Common Interpretive Anchor	Contextual Warning	Source Context
Blood lactate	Resting values are often ~0.5–2.0 mmol·L^−1^; fixed 4 mmol·L^−1^ thresholds are historically used but not individualized [37,38,150]	Interpret with workload, protocol, nutrition, glycogen status, sampling site, and training status	Exercise-specific
CK	Athlete-specific reference intervals reported as 82–1083 U/L in male athletes and 47–513 U/L in female athletes [9,61,149]	High CK alone does not diagnose injury, overtraining, or rhabdomyolysis	Exercise-specific
LDH	Adult laboratory ranges commonly vary around ~120–250 U/L, but assays differ [8,62]	Nonspecific; hemolysis, liver, heart, erythrocytes, and muscle can contribute	General adult/clinical
Myoglobin	Often low at rest, commonly <70–100 ng·mL^−1^, but method-dependent [62,63]	Early and rapidly cleared; interpret with CK, urine, creatinine, symptoms, and timing	General adult/clinical
Sodium	Exercise-associated hyponatremia: Na^+^ < 135 mmol·L^−1^ during or up to 24 h after exercise [70,71]	Symptoms and fluid history determine urgency; do not treat all collapse as dehydration	Exercise-specific
Potassium	Typical adult serum range is commonly ~3.5–5.0 mmol·L^−1^ [63,65,70,71]	Hemolysis, acidosis, renal function, rhabdomyolysis, and timing can distort interpretation	General adult/clinical
Creatinine	Common adult laboratory ranges are roughly ~0.6–1.3 mg·dL^−1^, but muscular athletes may sit higher without renal disease [7,8,72,73]	Interpret with muscle mass, creatine use, hydration, CK, eGFR, urine findings, and exercise timing	Mixed evidence
BUN	Common clinical orientation range is often ~7–20 mg·dL^−1^ [8,69,71,74]	High protein intake, dehydration, energy deficit, and endurance volume can all increase BUN	General adult/clinical
Urea	Common serum urea orientation ranges are often ~2.5–7.1 mmol·L^−1^, depending on laboratory conversion and method [8,65,67]	Interpret with BUN units, protein intake, hydration, renal function, and carbohydrate availability	General adult/clinical
Hemoglobin	Common clinical anemia thresholds are approximately <13 g·dL^−1^ in men and <12 g·dL^−1^ in women, but athlete plasma volume matters [80,81,82,83]	Low Hb may reflect pseudoanemia, iron deficiency, illness, or true anemia	Mixed evidence
Hematocrit	Common adult intervals are often ~40–52% in men and ~36–46% in women [80,82,83]	Lower Hct in endurance athletes may reflect plasma volume expansion rather than anemia	Mixed evidence
Ferritin	Low ferritin often suggests depleted stores; <15–30 µg·L^−1^ is commonly concerning, while athlete targets may be higher in endurance/altitude contexts [11,80,84,85]	Ferritin rises with inflammation; interpret with CRP, TSAT, sTfR, and symptoms	Mixed evidence
TSAT	Low TSAT, often <16–20%, may suggest restricted circulating iron availability [80,84,86,87]	Interpret with ferritin, CRP, transferrin/TIBC, serum iron, and symptoms	General adult/clinical
sTfR	Elevated sTfR suggests cellular iron demand or iron-deficient erythropoiesis [86,87]	Assay-dependent; useful when ferritin is confounded by inflammation	General adult/clinical
25(OH)D	<50 nmol·L^−1^ is commonly considered deficient/inadequate, whereas 50–75 nmol·L^−1^ is often considered insufficient [96]. Athlete literature has used heterogeneous classifications, including thresholds around 75–80 nmol·L^−1^ to define sufficiency or inadequacy [95,169]	Classification thresholds are not validated universal athlete performance targets; interpret with season, sun exposure, supplementation, calcium/PTH, LEA/REDs risk, bone-stress history, assay context, and toxicity risk.	Mixed evidence
CRP/hs-CRP	<1, 1–3, and >3 mg·L^−1^ are often used as low, moderate, and high cardiovascular-risk categories [102,103,173]	In athletes, recent exercise, infection, muscle damage, or competition may dominate interpretation	General adult/clinical
Cortisol	Morning serum values are commonly much higher than evening values; approximate morning ranges often sit around ~5–25 µg·dL^−1^, assay-dependent [119,120,122]	General clinical orientation only, not an athlete-specific range; strong circadian, sleep, travel, stress, illness, and matrix dependence	General adult/clinical
Testosterone	In men, consistently low morning total testosterone, often around <264–300 ng·dL^−1^, requires symptoms and repeat testing [124,174]	General clinical orientation only, not an athlete-specific performance threshold; interpret with SHBG, free testosterone, energy availability, sleep, illness, and training history	General adult/clinical
Thyroid-stimulating hormone (TSH)	Adult reference intervals often cluster around ~0.4–4.0 mIU·L^−1^, but method and population vary [17,119,128,129]	General clinical orientation only; low T3 with normal TSH may reflect LEA-related metabolic adaptation rather than primary thyroid disease	General adult/clinical
β-hydroxybutyrate	Nutritional ketosis often begins around ≥0.5 mmol·L^−1^, but exercise/fasting context matters [78,79]	Elevated values can reflect fasting, low carbohydrate availability, exogenous ketones, prolonged exercise, or LEA context	Mixed evidence
Urine specific gravity	Values >1.020–1.025 are often used as practical markers of concentrated urine/hypohydration risk [70,71]	Recent fluid intake, timing, renal handling, and sweat losses strongly influence values	Exercise-specific

## Data Availability

No new data were created or analyzed in this study. Data sharing is not applicable to this article.

## Abbreviations

The following abbreviations are used in this manuscript:

1,25(OH)_2_D	1,25-dihydroxyvitamin D
25(OH)D	25-hydroxyvitamin D
ADP	Adenosine diphosphate
AHR	Aryl hydrocarbon receptor
ALT	Alanine aminotransferase
AMP	Adenosine monophosphate
AST	Aspartate aminotransferase
ATP	Adenosine triphosphate
ATP-ase	Adenosine triphosphatase
BAIBA	β-aminoisobutyric acid
BALP	Bone-specific alkaline phosphatase
BASE	Baseline, Analytical standardization, Sport-specific context, and Evidence of functional change
BUN	Blood urea nitrogen
Ca^2+^	Calcium ion
CHO	Carbohydrate
CK	Creatine kinase
CK-MM	Skeletal muscle creatine kinase isoenzyme
Cl^-^	Chloride ion
CoA	Coenzyme A
CRP	C-reactive protein
CTX-I	C-terminal telopeptide of type I collagen
Dcytb	Duodenal cytochrome b
Dmax	Maximal distance method (Dmax)
DMT1	Divalent metal transporter 1
DOMS	Delayed-onset muscle soreness
eGFR	Estimated glomerular filtration rate
EIMD	Exercise-induced muscle damage
EPO	Erythropoietin
FATmax	Exercise intensity eliciting maximal fat oxidation
Fe^2+^	Ferrous iron
Fe^3+^	Ferric iron
FPN	Ferroportin
FFA	Free fatty acid
FSH	Follicle-stimulating hormone
FXR	Farnesoid X receptor
GC-MS	Gas chromatography–mass spectrometry
GH	Growth hormone
GPS	Global positioning system
H^+^	Hydrogen ion
HAMP	Hepcidin antimicrobial peptide
Hb	Hemoglobin
HCO_3_^−^	Bicarbonate
Hct	Hematocrit
HFE	Homeostatic iron regulator
HIIT	High-intensity interval training
HMDB	Human Metabolome Database
HPA	Hypothalamic–pituitary–adrenal
HPG	Hypothalamic-pituitary-gonadal
HR	Heart rate
HRV	Heart rate variability
hs-CRP	High-sensitivity C-reactive protein
IGF-1	Insulin-like growth factor-1
IL-1β	Interleukin-1 beta
IL-6	Interleukin-6
IL-8	Interleukin-8
IL-10	Interleukin-10
K^+^	Potassium ion
KIM-1	Kidney injury molecule-1
Lac-Phe	N-lactoyl-phenylalanine
LC-MS	Liquid chromatography–mass spectrometry
LDH	Lactate dehydrogenase
LEA	Low energy availability
LH	Luteinizing hormone
MCH	Mean corpuscular hemoglobin
MCT1	Monocarboxylate transporter 1
MCT4	Monocarboxylate transporter 4
MCV	Mean corpuscular volume
MFO	Maximal fat oxidation
Mg^2+^	Magnesium ion
MLSS	Maximal lactate steady state
MoTrPAC	Molecular Transducers of Physical Activity Consortium
mTOR	Mechanistic target of rapamycin
Na^+^	Sodium ion
NAD^+^	Nicotinamide adenine dinucleotide, oxidized form
NADH	Nicotinamide adenine dinucleotide, reduced form
NEFA	Non-esterified fatty acids
NGAL	Neutrophil gelatinase-associated lipocalin
NH_3_	Ammonia
NH_4_^+^	Ammonium ion
NIH	National Institutes of Health
NMR	Nuclear magnetic resonance
OC	Osteocalcin
PCr	Phosphocreatine
PGC-1α	Peroxisome proliferator-activated receptor gamma coactivator 1-alpha
PGC-1α1	Peroxisome proliferator-activated receptor gamma coactivator 1-alpha 1
Pi	Inorganic phosphate
P1NP	Procollagen type I N-terminal propeptide
PTH	Parathyroid hormone
REDs	Relative Energy Deficiency in Sport
RER	Respiratory exchange ratio
ROS	Reactive oxygen species
RNS	Reactive nitrogen species
RPE	Rating of perceived exertion
SCFA	Short-chain fatty acid
SCFAs	Short-chain fatty acids
SHBG	Sex hormone-binding globulin
STAT3	Signal transducer and activator of transcription 3
sTfR	Soluble transferrin receptor
T3	Triiodothyronine
T4	Thyroxine
TCA	Tricarboxylic acid
T:C ratio	Testosterone-to-cortisol ratio
TfR1	Transferrin receptor 1
TGR5	Takeda G protein-coupled receptor 5
TIBC	Total iron-binding capacity
TNF-α	Tumor necrosis factor-alpha
TSAT	Transferrin saturation
TSH	Thyroid-stimulating hormone
VO_2_	Oxygen uptake
W′	Work capacity above critical power
β-hydroxybutyrate	Beta-hydroxybutyrate
β-oxidation	Beta-oxidation
